# Structural insights into tyrosine sulfation of CCR5 by human tyrosylprotein sulfotransferase‐1

**DOI:** 10.1111/febs.70597

**Published:** 2026-05-22

**Authors:** Shinnosuke Tanaka, Hirai Asano, Kota Toyoda, Toshiaki Nishiyori, Hidetaka Kojo, Kazuto Kiyomatsu, Katsuhisa Kurogi, Yoichi Sakakibara, Etsuko Nishimoto, Takamasa Teramoto, Yoshimitsu Kakuta

**Affiliations:** ^1^ Faculty of Agriculture, Laboratory of Biophysical Chemistry, Department of Bioscience and Biotechnology Kyushu University Fukuoka Japan; ^2^ Faculty of Agriculture, Department of Applied Biochemistry and Biotechnology University of Miyazaki Miyazaki Japan

**Keywords:** CCR5, crystal structure, structural modeling, tyrosine sulfation, tyrosylprotein sulfotransferase

## Abstract

Post‐translational sulfation of the chemokine receptor CCR5 is involved in crucial biological processes such as viral infection and chemokine signaling. This sulfation can occur at the N‐terminal tyrosine residues (Y3, Y10, Y14, and Y15) of CCR5 and is catalyzed by human tyrosylprotein sulfotransferases (hTPSTs) within the Golgi lumen. However, the detailed molecular mechanism by which these tyrosine residues are sulfated remains unresolved. To elucidate the mechanism, we determined the crystal structure of a soluble domain of hTPST1 bound to the sulfate donor product 3′‐phosphoadenosine 5′‐phosphate (PAP) and a modified 18‐residue CCR5 peptide designed to isolate the Y3‐centered binding mode, at 3.2 Å resolution, with six peptide residues ordered. This structure defines key interactions consistent with Y3 sulfation and is consistent with previous biochemical data. Based on the crystal structure and prior knowledge, we constructed peptide docking models for Y10, Y14, and Y15 sulfation, as well as full‐length hTPST1‐PAP‐CCR5 docking models. The crystal structure provides experimental insight into Y3 recognition, whereas the docking models provide testable hypotheses for how the other sulfation sites may be accommodated in the context of CCR5.

## Graphical abstract

Post‐translational sulfation of the chemokine receptor CCR5 is involved in crucial biological processes such as viral infection and chemokine signaling. This sulfation is catalyzed by human tyrosylprotein sulfotransferases (hTPSTs) within the Golgi lumen. However, the detailed molecular mechanism remains unresolved. Structural analysis of hTPST1 bound to a CCR5 N‐terminal peptide reveals how hTPST1 recognizes the Tyr3 sulfation site. Structure‐guided models of additional CCR5 sulfation states and full‐length assemblies provide a framework for understanding CCR5 tyrosine sulfation, a post‐translational modification relevant to viral entry and chemokine signaling.

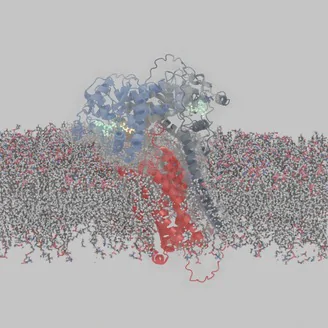

**Abbreviations**
CCR5: C‐C chemokine receptor type 5
hTPST: human tyrosylprotein sulfotransferase
hTPST1: human tyrosylprotein sulfotransferase‐1
hTPST2: human tyrosylprotein sulfotransferase‐2
PAP: 3′‐phosphoadenosine 5′‐phosphate
PAPS: 3′‐phosphoadenosine 5′‐phosphosulfate
RMSD: root‐mean‐square deviation
SASA: solvent‐accessible surface area
TM: transmembrane

## Introduction

Tyrosine sulfation is a widespread post‐translational modification that enhances protein–protein interactions and is crucial for numerous physiological processes, including immune responses, cellular signaling, and pathogenic viral or microbial infections [1, 2, 3, 4, 5, 6]. Tyrosine sulfation occurs in secreted and membrane proteins within the Golgi lumen, catalyzed by human tyrosylprotein sulfotransferases (hTPSTs) (Fig. 1A) [7]. For example, chemokine receptors, PSGL‐1, and the C5a receptor are known tyrosine‐sulfated membrane proteins [2, 4, 8, 9]. hTPSTs sulfate the extracellular domains of substrates, and the resulting sulfated tyrosine residues strengthen protein–protein interactions [9]. Chemokine receptors are classified into several subfamilies (CXCR, CCR, XCR, and CX3CR) [10, 11]. Sequence variations among these receptors, including sulfated tyrosine residues in the flexible N‐terminal region, confer specificity for chemokine ligand binding [12, 13] (Fig. S1).

**Fig. 1:**
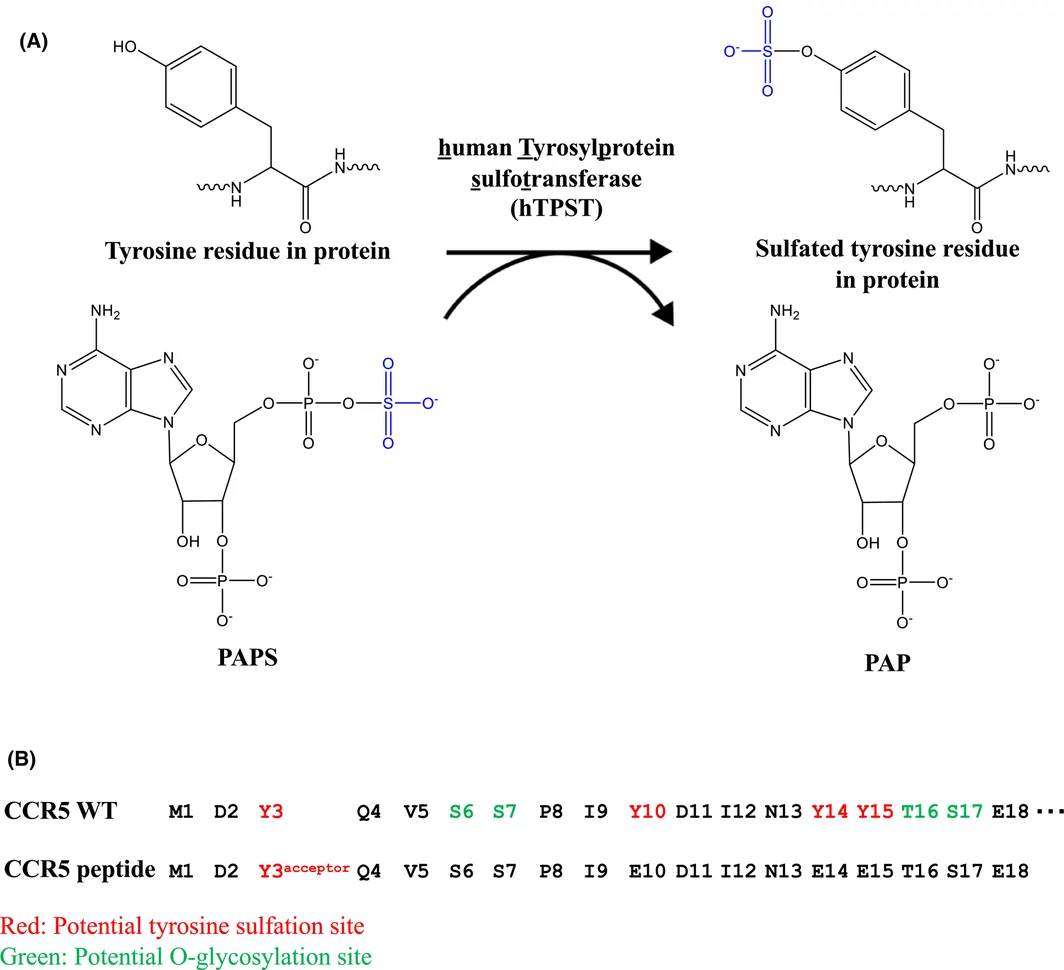
Tyrosine sulfation reaction catalyzed by hTPSTs and CCR5 tyrosine sulfation sites. (A) Tyrosine sulfation reaction scheme catalyzed by human tyrosylprotein sulfotransferases (hTPSTs). (B) The amino acid sequence of the wild‐type CCR5 N‐terminal region is shown in the upper row. The 18‐amino acid sequence of the CCR5 peptide derived from the CCR5 N‐terminal region is shown in the lower row. The CCR5 peptide was designed to be centered on Y3; the three other tyrosine residues (Y10, Y14, and Y15) were substituted with glutamic acid.

Among these receptor families, CCR5 stands out because of its pivotal roles in ligand‐mediated immune responses, viral infection, and microbial toxin recognition [12, 14]. The N‐terminal extracellular domain of CCR5 contains a flexible region that mediates ligand interactions, and its tyrosine residues (Y3, Y10, Y14, and Y15) are sulfated by hTPSTs in the Golgi lumen [8]. This N‐terminal region also contains four potentially O‐glycosylated serine/threonine residues, and the resulting heterogeneous sulfation and glycosylation states generate clusters of negative charge that are essential for receptor function [15] (Fig. 1B; Fig. S1). Sulfation at Y10, Y14, and Y15 is critical for binding of the HIV‐1 gp120 envelope glycoprotein [8] and sulfated CCR5 functions together with CD4 as a coreceptor for HIV entry [12, 16, 17, 18, 19, 20]. Recently, an antibody targeting the sulfated CCR5 N‐terminus was investigated as a potential tool for anti‐HIV drug discovery [21]. Sulfation at Y10, Y14, and Y15 enhances binding to the chemokine CCL5, whereas sulfation at Y3 only modestly increases CCL5 binding [12, 22]. In addition, a CCR5 peptide containing Y3 shows increased binding to the noncognate chemokine CXCL12 compared with the CCR5 8–20 peptide [23]. CCR5 can potentially bind multiple chemokines, including CCL3, CCL3L1, CCL4, CCL5, CCL4L1, CCL8, CCL11, and CCL16, although not all of these interactions have been fully characterized [11]. Sulfation at Y3 has been particularly difficult to study because of the difficulty of synthesizing triply or quadruply sulfated peptides and because Y3 is distal to the other tyrosine residues. In addition, the N‐terminal modifications of CCR5 generate ‘glyco‐sulfo barcodes’ that may confer specific ligand‐binding properties [15]. With respect to CCR5 tyrosine sulfation, mass spectrometry studies showed that hTPST2 preferentially sulfates Y3 in the CCR5 1–18 peptide, which includes the N‐terminal methionine [24], whereas Y3 is the last residue to be sulfated in the CCR5 2–18 peptide by both hTPST1 and hTPST2 [25]. For hTPST1, however, the sulfation order of the CCR5 1–18 peptide has not yet been established experimentally. Nevertheless, the highly similar substrate‐binding pockets observed in the previously reported crystal structures of hTPST1 and hTPST2 [26, 27] suggest that hTPST1 could accommodate the M1‐containing CCR5 peptide in a similar Y3‐centered binding mode. Y3 is also considered a favorable sulfation site because the residue immediately N‐terminal to Y3 is aspartic acid, which has been identified as a major determinant of the hTPST *K*_*m*_ [27, 28].

hTPSTs catalyze the sulfation of protein tyrosine residues within the Golgi apparatus [3]. These enzymes transfer the sulfonate group from 3′‐phosphoadenosine 5′‐phosphosulfate (PAPS) to protein tyrosine residues in the Golgi lumen, yielding sulfated tyrosine substrates and 3′‐phosphoadenosine 5′‐phosphate (PAP) (Fig. 1A). In humans, there are two TPST isoforms, hTPST1 and hTPST2, which share 64% amino acid sequence identity [29, 30]. Both are type II transmembrane proteins comprising an N‐terminal short intracellular domain, a 17‐residue transmembrane helix, a 40‐residue stem domain, and a C‐terminal catalytic domain [29, 30]. They are also capable of forming heterodimers [31]. Although crystal structures of hTPST‐substrate complexes (e.g., complement C4‐derived peptide and gastrin‐derived peptide) have been solved [26, 27], these data alone are insufficient to explain the molecular mechanism of tyrosine sulfation in the CCR5 N‐terminal domain. In our previous work, we solved the crystal structures of hTPST2‐PAP‐C4 peptide (C4P5Y3 peptide), hTPST1‐PAP‐C4 peptide (C4P5Y5 peptide), and hTPST1‐PAP‐gastrin peptide, revealing the basic sulfation mechanism [26, 27]. These structures suggested that hTPST1 and hTPST2 share nearly identical catalytic residues and substrate‐recognition modes, and that they can form a structural heterodimer. hTPSTs recognize various substrates using hydrophobic and electrostatic interactions. Studies of TPSTs from other organisms (e.g., ticks) further demonstrate sequential sulfation of multiple tyrosine residues, highlighting the conserved nature of TPST mechanisms [32]. In this way, the basic mechanism of tyrosine sulfation by TPSTs has gradually been clarified.

Despite the biological importance of CCR5 N‐terminal tyrosine sulfation, its detailed molecular mechanism remains unresolved. Here, we present the crystal structure of the hTPST1‐PAP‐CCR5 peptide complex, revealing a Y3‐centered recognition mode and interactions associated with Y3 sulfation. Structural analysis and comparison indicated that the observed hTPST1–CCR5 interface is most consistent with contributions from the ordered M1, D2, and Y3 residues. To explore how all four tyrosine residues of CCR5 may be sulfated, we also developed structure‐guided models for the other sulfation states and for full‐length hTPST1‐CCR5 assemblies [33]. These analyses provide a structural framework for future mechanistic studies of CCR5 tyrosine sulfation and for hypothesis‐driven evaluation of how CCR sulfation may be modulated in viral infection and chemokine signaling. These full‐length assemblies are presented as structure‐guided docking models for hypothesis generation and future experimental testing.

## Results and Discussion

### Crystal structure of hTPST1‐PAP‐CCR5 peptide complex

Y3 is considered the preferred sulfate acceptor among the four tyrosine residues of CCR5 when the N‐terminal methionine is present, according to previous mass spectrometry data reported for hTPST2 [24]. While this preference has not been experimentally established for hTPST1, our structure indicates that hTPST1 can accommodate the M1‐containing CCR5 peptide in a Y3‐centered binding mode. Thus, we prepared a modified CCR5 peptide designed to isolate the Y3‐centered binding mode for crystallographic analysis (sequence: MDYQVSSPIEDINEETSE, in which Y10, Y14, and Y15 were substituted with glutamic acid to suppress alternative tyrosine‐centered binding modes) and measured sulfotransferase activity toward this peptide using the soluble domain of hTPST1. The kinetic assay yielded a *K*_m_ of 7.32 × 10^2^ μM and a *k*_cat_/*K*_m_ of 1.40 × 10^−5^ min^−1^·μM^−1^ (Table 1). Previous reports showed that an aspartic acid residue at the −1 position and an acidic residue at the +1 position are major determinants of the *K*_m_ of hTPST2 [26, 28]. Both residues are present in the C4 peptide but absent from the gastrin peptide, whereas the CCR5 peptide contains only the aspartic acid residue at the −1 position (Table 1 and Fig. S2). Consequently, the *K*_m_ for the CCR5 peptide was higher than that for the C4 peptide and similar to that of the gastrin peptide. Also, the catalytic efficiency (*k*_cat_/*K*_m_) of the CCR5 peptide was 456‐fold lower than that of the C4 peptide and slightly higher than that of the gastrin peptide.

**Table 1:** Kinetic parameters of hTPST1 with substrate peptides.

Substrate	*K*_m_ (μM)	*k*_cat_ (min^−1^)	*k*_cat_/*K*_m_ (min^−1^·μM^−1^)
CCR5 peptide	7.32 × 10^2^ ± 6.9 × 10	1.03 × 10^−2^ ± 2.2 × 10^−4^	1.40 × 10^−5^
C4 peptide*	7.67 ± 1.7	4.90 × 10^−2^ ± 2.4 × 10^−3^	6.39 × 10^−3^
gastrin peptide*	6.52 × 10^2^ ± 4.1 × 10	6.57 × 10^−3^ ± 4.5 × 10^−4^	1.01 × 10^−5^

* C4 peptide and gastrin peptide kinetic parameters were previously reported [26].

To gain deeper insight into the molecular mechanisms underlying the observed kinetic properties, we next performed crystallographic analysis of the hTPST1‐PAP‐CCR5 peptide complex. We solved the crystal structure of the hTPST1‐PAP‐CCR5 peptide complex at 3.2 Å resolution. The data collection and refinement statistics are summarized in Table 2. The refined model contains electron density consistent with two metal‐binding sites, which were modeled as Mg^2+^ and Zn^2+^ for refinement (Table 2). Although these ions were not added to the storage buffer or crystallization reservoir, they may have been carried through purification and retained in the protein sample. Their identities remain unconfirmed in the absence of anomalous diffraction data or elemental analyses. The overall structure shows an hTPST1 dimer bound to two PAP molecules and two CCR5 peptides (Fig. 2A). The asymmetric unit contains eight hTPST1 molecules, organized into four dimers (A/B, C/D, E/F, and G/H). Of the 18 CCR5 peptide residues, six residues (M1, D2, Y3, Q4, V5, and S6) were ordered in three of the eight molecules based on the electron density maps. In the remaining five molecules, five residues (M1, D2, Y3, Q4, and V5) of the CCR5 peptide were determined (Fig. 2B,C). Superposition of the four hTPST1 dimers (A/B, C/D, E/F, and G/H) yielded RMSDs of 0.24–0.34 Å over 551–552 atoms, indicating that the dimeric arrangement is also highly conserved within the asymmetric unit (Fig. S3A). In addition, superposition of the eight hTPST1 protomers yielded root‐mean‐square deviations (RMSDs) of 0.15–0.31 Å over 272 Cα atoms, and the bound CCR5 peptides showed RMSDs of 0.35–0.88 Å over five Cα atoms (Fig. S3B,C). The overall conformations of hTPST1 were highly similar, and the CCR5 peptide adopted a conserved binding mode across the eight protomers, although variations in the C‐terminal region were observed. Therefore, we used hTPST1 protomer‐A with the bound CCR5 peptide as the representative model. The hTPST1 dimer presents two deep substrate‐binding pockets, each accommodating one CCR5 peptide (Fig. 2D). Minor differences among the bound CCR5 peptides were observed mainly at the C‐terminal end, whereas the M1–V5 segment adopted a similar recognition mode across the asymmetric unit.

**Table 2:** Crystallographic data collection and refinement statistics. Metal identities are provisional and were used only for refinement; they were not confirmed by anomalous diffraction or elemental analysis. Structure was determined from a single crystal. Values in parentheses are for highest‐resolution shell. hTPST1, human tyrosylprotein sulfotransferase‐1; PAP, 3′‐phosphoadenosine‐5′‐phosphate.

	hTPST1‐PAP‐CCR5‐peptide
Data collection	
Space group	*C*2
Cell dimensions	
*a*, *b*, *c* (Å)	188.9, 189.4, 121.5
*α*, *β*, *γ* (°)	90.0, 126.0, 90.0
Resolution (Å)	50.0–3.20 (3.26–3.20)*
*R*_merge_	0.555 (0.990)
CC1/2	0.990 (0.484)
*I*/σ*I*	2.73 (0.98)
Completeness (%)	99.9 (99.6)
Redundancy	3.7 (3.1)
Refinement	
Resolution (Å)	49.21–3.20
No. reflections	56 568
*R*_work_/*R*_free_	0.273/0.323
No. atoms	
Protein	17 544
Ligand (PAP)	216
Ligand (Peptide)	370
Putative Mg^2+^	8
Putative Zn^2+^	4
*B*‐factors	
Protein	23.4
Ligand (PAP)	14.0
Ligand (Peptide)	45.4
Putative Mg^2+^	12.9
Putative Zn^2+^	78.4
R.m.s. deviations	
Bond lengths (Å)	0.004
Bond angles (°)	0.771
Ramachandran plot	
Favored (%)	98.06
Allowed (%)	1.94
Disallowed (%)	0.00

* Values in parentheses are for the highest‐resolution shell.

**Fig. 2:**
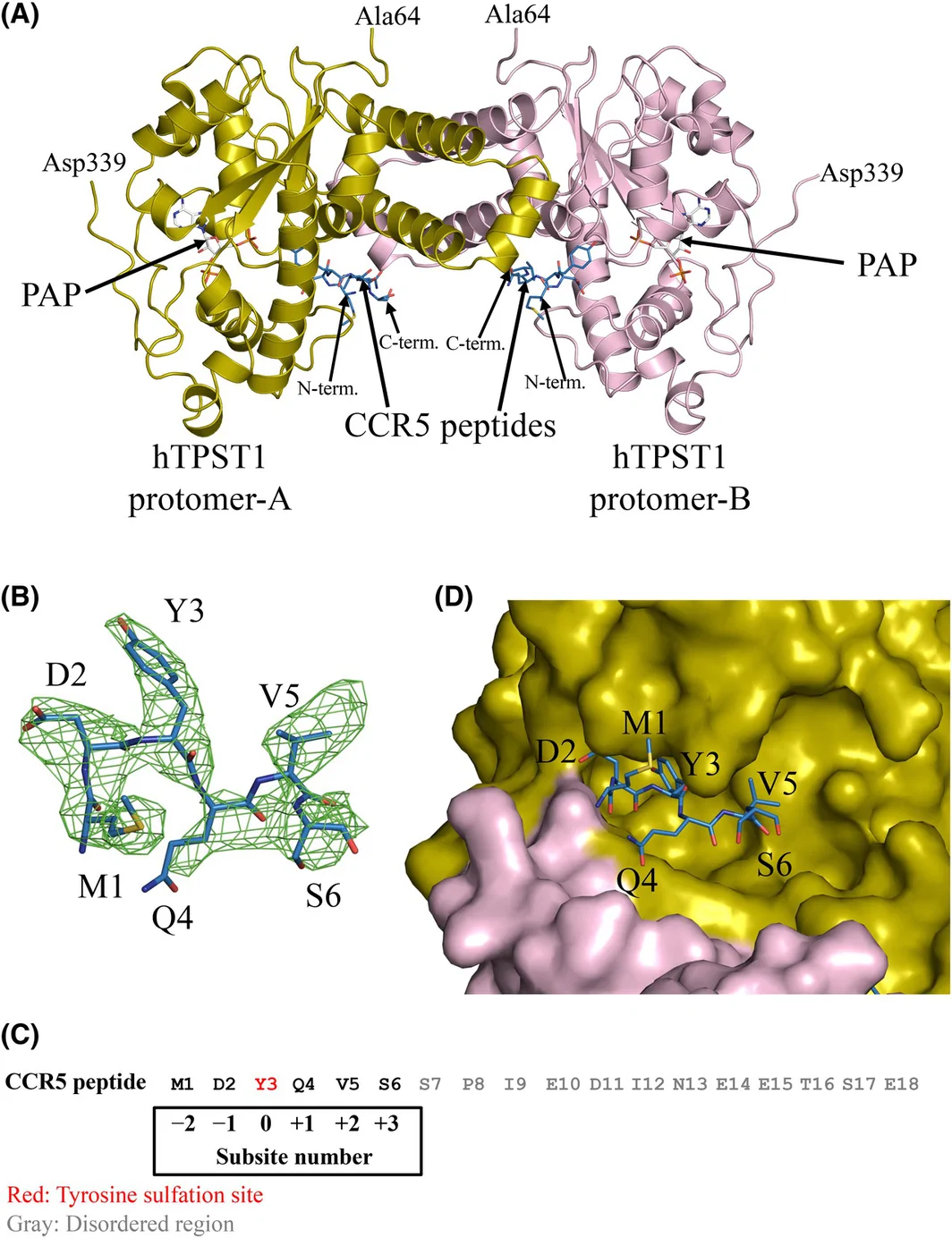
Overall structure of the hTPST1‐PAP‐CCR5 peptide complex. (A) Overall ribbon model structure of the hTPST1‐PAP‐CCR5 peptide complex. Protomer‐A and protomer‐B of hTPST1 are colored olive and light pink, respectively. The CCR5 peptide and PAP are colored sky blue and white, respectively, and are depicted as stick models. To distinguish the sequences, hTPST1 residues are shown using the three‐letter code, whereas CCR5 residues are shown using the one‐letter code. (B) Polder omit electron density map (green, contoured at 3.4 σ) showing the CCR5 peptide bound to hTPST1 protomer‐A. (C) Ordered CCR5 peptide residues are shown in black, whereas disordered residues are shown in gray. Subsite numbers are indicated below the sequence, with the acceptor tyrosine residue defined as position 0. (D) hTPST1 surface model with the CCR5 peptide shown as a stick model. The structural models shown in panels A, B, and D are based on the X‐ray crystal structure of the hTPST1‐PAP‐CCR5 peptide complex determined in this study. Molecular graphics were prepared using PyMOL.

The crystal structure revealed that hTPST1 forms a dimer with the same overall architecture, consistent with the previously reported hTPST1‐PAP‐C4 peptide (PDB ID: 5WRI) and hTPST1‐PAP‐gastrin peptide (PDB ID: 5WRJ) structures. Protomer‐A of the hTPST1‐PAP‐CCR5 peptide complex superimposes on that of the hTPST1‐PAP‐C4 peptide complex with an RMSD of 0.37 Å over 271 Cα atoms (Fig. S4A). Likewise, protomer‐A of the hTPST1‐PAP‐CCR5 peptide complex superimposes on that of the hTPST1‐PAP‐gastrin peptide complex with an RMSD of 0.37 Å over 271 Cα atoms (Fig. S4B). These results indicate that the overall hTPST1 structure is essentially the same.

### Mechanism of CCR5 Y3 sulfation by hTPST1

Previous mass spectrometry analysis of the CCR5 1–18 peptide showed that Y3 is the preferred sulfate acceptor for hTPST2 among the four tyrosine residues [24]. Conversely, a study of the CCR5 2–18 peptide by Seibert *et al*. demonstrated that Y3 was the final residue to be sulfated by both hTPST1 and hTPST2 [25]. Our crystal structure now reveals the molecular basis by which hTPST1 recognizes the Y3‐centered segment of CCR5. In the hTPST1‐PAP‐CCR5 peptide crystal structure, the CCR5 peptide forms a parallel β‐sheet with hTPST1 Thr199‐Ala201, consistent with previous structural studies (Fig. 3A). This finding supports the notion that parallel β‐sheet formation is important for hTPST enzymes to accommodate a variety of substrate peptides, as shown previously [26, 32].

**Fig. 3:**
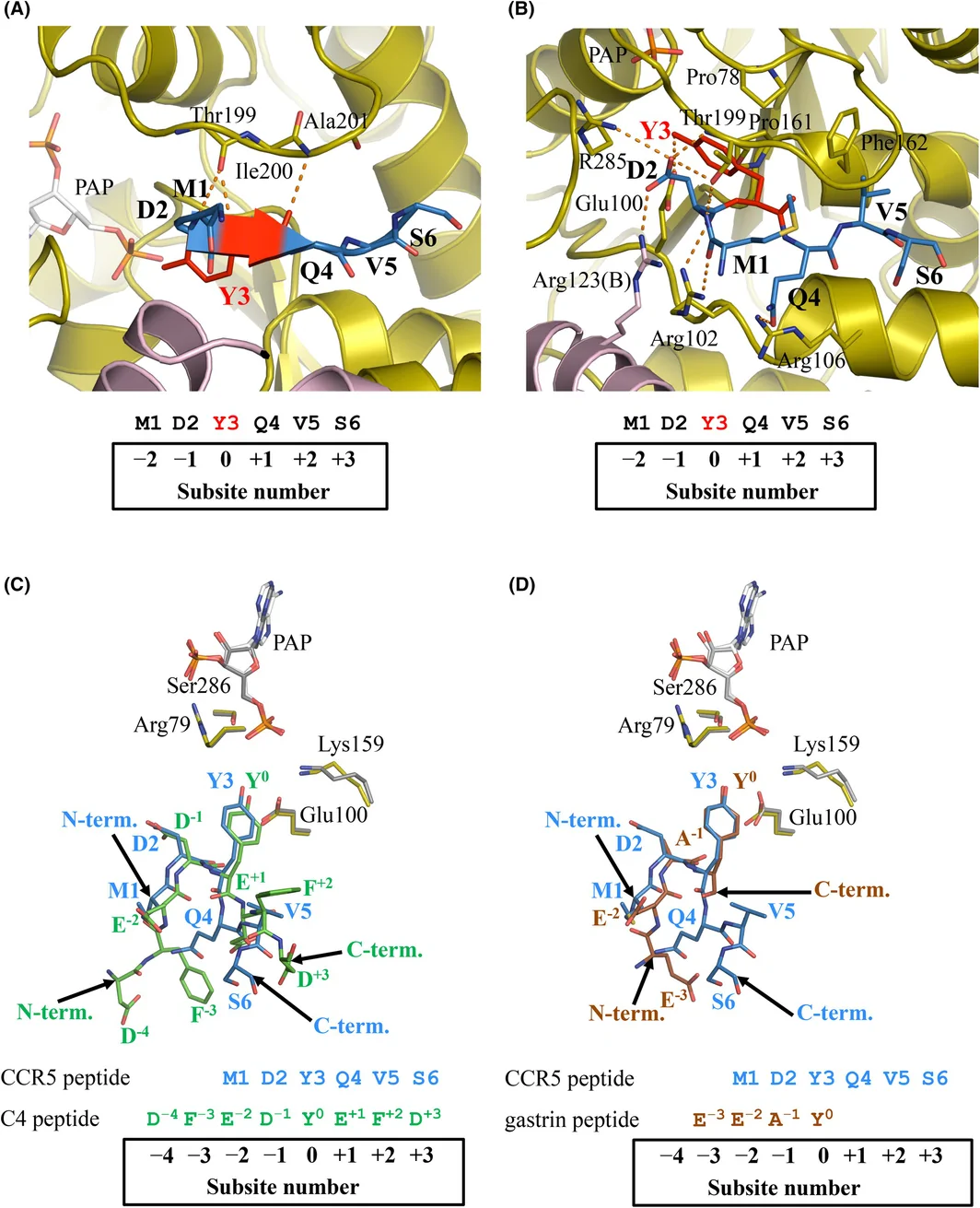
Interactions between the CCR5 peptide and hTPST1, and the structural superposition of three substrate–peptide complexes. (A) Close‐up view of the parallel β‐sheet interaction of hTPST1 and the CCR5 peptide. (B) Close‐up view of interactions between hTPST1 and the CCR5 peptide other than the β‐sheet. Arg123 (B) denotes Arg123 from hTPST1 protomer‐B. (C) Superposition of the hTPST1‐PAP‐C4 peptide and the hTPST1‐PAP‐CCR5 peptide. The C4 peptide and the CCR5 peptide are colored in green and sky blue, respectively. In the hTPST1‐PAP‐C4 peptide, PAP and the catalytic residues of hTPST1 are shown as gray sticks. In the hTPST1‐PAP‐CCR5 peptide, PAP is shown in white sticks, and the catalytic residues of hTPST1 are shown in olive sticks. (D) Superposition of the hTPST1‐PAP‐gastrin peptide and the hTPST1‐PAP‐CCR5 peptide. The gastrin peptide and the CCR5 peptide are colored in brown and sky blue, respectively. The gastrin peptide includes nonproductive complex and productive complex, and here only the productive gastrin peptide binding mode is shown for comparison. In the hTPST1‐PAP‐gastrin peptide, PAP and the catalytic residues of hTPST1 are shown in gray sticks. The structural models shown in panels A and B are based on the X‐ray crystal structure of the hTPST1‐PAP‐CCR5 peptide complex determined in this study. The structural models shown in panel C are based on the X‐ray crystal structure of the hTPST1‐PAP‐CCR5 peptide complex determined in this study and the previously reported X‐ray crystal structure of the hTPST1‐PAP‐C4 peptide complex [27]. The structural models shown in panel D are based on the X‐ray crystal structure of the hTPST1‐PAP‐CCR5 peptide complex determined in this study and the previously reported X‐ray crystal structure of the hTPST1–PAP–gastrin peptide complex [27]. Structural superpositions were performed using Coot [34], and molecular graphics were prepared using PyMOL.

The sequence differences between the CCR5 peptide and the C4 peptide, which is the longest defined substrate peptide, are M1 versus E^−2^ at the −2 position, Q4 versus E^+1^ at the +1 position, V5 versus F^+2^ at the +2 position, and S6 versus D^+3^ at the +3 position in the determined structures. The main‐chain carbonyl group of M1 is positioned in proximity to Arg102 (Fig. 3B). Arg102 is conserved between hTPST1 and hTPST2. The M1 side chain appears to be partially buried upon complex formation. To evaluate this, we calculated the solvent‐accessible surface area (SASA) of the M1 side chain. The SASA was 73.3 Å^2^ in the hTPST1‐bound state and 143.6 Å^2^ in the isolated state, indicating that approximately half of the side‐chain surface is buried upon complex formation. These data support the view that the M1 side chain contributes to the binding interface of hTPST1. As noted above, the sulfation order of the CCR5 1–18 peptide has been established for hTPST2 but has not yet been experimentally established for hTPST1. Given that hTPST1 and hTPST2 possess highly conserved substrate‐binding pockets (Fig. S5A,B), our structure suggests that hTPST1 can accommodate the M1‐containing CCR5 peptide in a Y3‐centered binding mode. However, the sulfation order of the CCR5 1–18 peptide remains to be determined experimentally for hTPST1.

D2 and Y3 make interactions with hTPST1 residues similar to those observed in the C4 peptide complex (Fig. S6). Specifically, the side chain of D2 is located near Arg123(B), Arg285, and Thr199. The main chain of D2 is positioned near Arg102 and Thr199. The hydroxyl group of Y3 is positioned near Glu100. Furthermore, the side chain of Q4 is located in the vicinity of Arg106. V5 is positioned near Phe162 of hTPST1, consistent with a possible hydrophobic interaction, similar to the F^+2^ residue of the C4 peptide. S6 faces away from the enzyme and does not appear to interact with any residues in hTPST1. Together, these observations indicate that the observed interface is most consistent with contributions from M1, D2, and Y3. Structural superpositions of three hTPST1 complexes show that the backbone of the acceptor tyrosine and its N‐terminal neighboring residues superimpose well, whereas the C‐terminal residues display greater variation (Fig. 3C,D). This implies that the acceptor tyrosine and its N‐terminal side residues are especially important for hTPST1 recognition. Consistent with this view, the absence of the N‐terminal M1 may contribute to the altered sulfation pattern observed for the CCR5 2–18 peptide, in which Y3 is sulfated last [25]. Moreover, while the tripeptide ADY has been proposed as a minimal consensus motif for efficient tyrosine sulfation [35], our results suggest that the interaction at the −2 position is primarily mediated by the peptide backbone. The absence of the consensus D residue at the −1 position for the Y10, Y14, and Y15 sites likely contributes to their lower catalytic efficiencies compared with Y3. Our crystal structure thus provides a structural basis for Y3 sulfation in CCR5 while showing that the hTPST1 binding pocket can accommodate more diverse sequence contexts with reduced efficiency. Although the density for the C‐terminal residues V5 and S6 was comparatively weak, the present chain tracing was supported by comparison with an alternative swapped model, which produced unfavorable geometry for V5 (Fig. S7A,B).

With regard to glycosylation, S6, S7, T16, and S17 are considered potential sites of sialylated O‐glycosylation (Fig. 1B) [22], functioning as glyco‐sulfo barcodes on the cell surface [15]. In the crystal structure, the side chain of S6 faces away from the hTPST1 binding pocket. We built two Y3 sulfation models of CCR5: one bearing S6‐GalNAc as a short glycan and another bearing S6‐GalNAc‐Gal‐Neu5Ac as a long glycan (Fig. S8A,B). In the sialylated O‐glycosylation pathway, GalNAc is first attached to the hydroxyl group of the serine side chain, and S6 does not interact with hTPST1 in our Y3 sulfation models. Based on these structural models, bulky glycans at S6 and S7 are unlikely to sterically hinder Y3 sulfation by hTPSTs, as these residues are oriented away from the enzyme's binding pocket in the Y3 recognition mode.

In summary, we elucidated the mechanism of Y3 sulfation in the CCR5 peptide from a structural biology perspective. Although the CCR5, C4, and gastrin peptides differ in sequence, hTPST1 accommodates each substrate. The CCR5 peptide forms a parallel β‐sheet with hTPST1, and the M1, D2, and Y3 residues make the major contributions to CCR5 binding. These detailed interactions are consistent with previously reported biochemical data [24, 35]. Previous reports also indicate that sulfated Y3 of CCR5 is critical for CXCL12 binding [23], plays a minor role in CCL5 binding [22], and that sulfation of Y3 or Y10 is required for binding of the anti‐CCR5 monoclonal antibody 3A9 to CCR5 [36]. Thus, Y3 sulfation contributes to biological function and may influence how the CCR5 N terminus is recognized by chemokines and antibodies. Our crystal structure provides a structural basis for CCR5 Y3 sulfation and a framework for future mechanistic and engineering studies.

### Docking models of hTPST1‐PAP‐CCR5 peptide for Y10, Y14, and Y15 sulfation

We have determined three different hTPST1‐PAP‐substrate complex structures. Although their overall folds and catalytic residues are similar, hTPST1 recognizes diverse substrate sequences through multiple interactions within the hTPST substrate‐binding pocket. To expand our insight into the sulfation mechanism at the other tyrosine residues, docking models of the hTPST1‐PAP‐CCR5 peptide for Y10, Y14, and Y15 sulfation were constructed based on the crystal structure of the hTPST1‐PAP‐CCR5 peptide centered on Y3 (Fig. 4A–D; Fig. S9A–D). In all models, the acceptor Y side chain is positioned near Glu100 for the catalytic sulfation reaction. To clarify the interactions between hTPST1 and the CCR5 peptide, we focused on the side‐chain interactions between the CCR5 peptide and hTPST1.

**Fig. 4:**
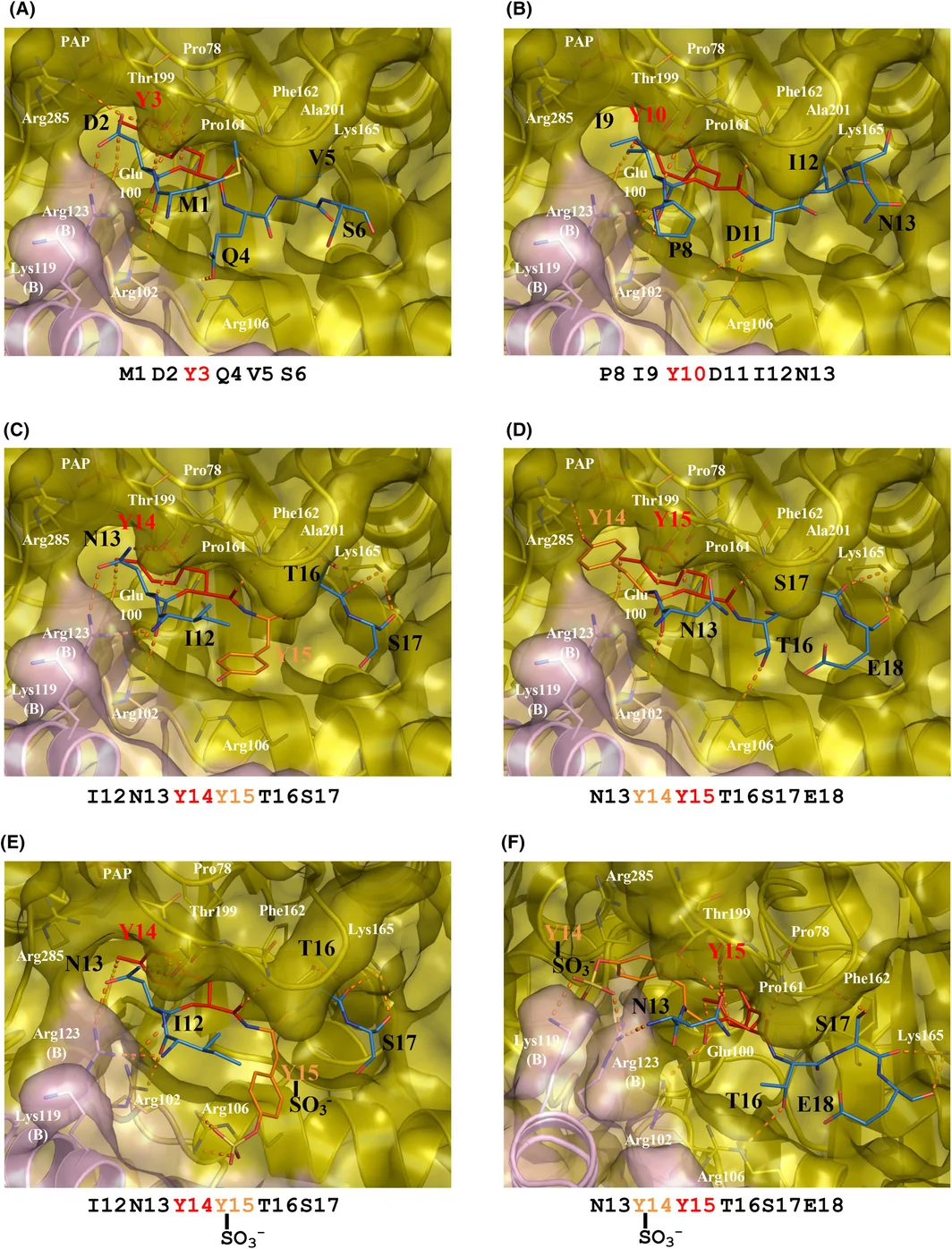
hTPST1‐PAP‐CCR5 peptide docking models for Y3, Y10, Y14, and Y15 sulfation. hTPST1 protomer‐A and protomer‐B are displayed as surface and ribbon models in olive and light pink, respectively. Key hTPST1 side chains are shown as stick models, and PAP is shown as a white stick model. Each CCR5 peptide is shown as a sky blue stick model. Acceptor tyrosine residues are colored red, while nonacceptor (other) tyrosine residues are colored orange. Dashed orange lines indicate putative proximity relationships in the modeled peptide conformations. (A) Crystal structure of the hTPST1–PAP–CCR5 peptide complex centered on Y3. (B) hTPST1‐PAP‐CCR5 peptide docking model for Y10 sulfation. (C) hTPST1‐PAP‐CCR5 peptide docking model for Y14 sulfation. (D) hTPST1‐PAP‐CCR5 peptide docking model for Y15 sulfation. (E) hTPST1‐PAP‐CCR5 peptide docking model with sY15 for Y14 sulfation. (F) hTPST1‐PAP‐CCR5 peptide docking model with sY14 for Y15 sulfation. sY14 and sY15 denote sulfated Y14 and sulfated Y15. See Fig. S9 for additional views. The hTPST1 structures shown in panels A–F are based on the X‐ray crystal structure of the hTPST1‐PAP‐CCR5 peptide complex determined in this study. In panel A, the CCR5 peptide corresponds to the crystallographically observed peptide. In panels B–E, the CCR5 peptides were modeled and energy‐minimized using the Molecular Operating Environment (MOE; Chemical Computing Group). In panel F, the CCR5 peptide and the side chain of Lys119 in protomer‐B were modeled and energy‐minimized using MOE. Molecular graphics were prepared using PyMOL.

In the hTPST1‐PAP‐CCR5 peptide docking model for Y10 sulfation, the modeled position of D11 of CCR5 places its side chain near Arg106 of hTPST1, consistent with a possible electrostatic interaction, and the modeled position of I12 lies near Phe162, consistent with a possible hydrophobic interaction. In the hTPST1‐PAP‐CCR5 peptide docking model for Y14 sulfation, the modeled position of N13 of CCR5 lies near Lys119(B) and Arg123(B) of hTPST1 protomer‐B. In addition, the modeled position of T16 is located near the main chain of Ala201, and the modeled position of S17 lies near Lys165. In the hTPST1‐PAP‐CCR5 peptide docking model for Y15 sulfation, the modeled position of Y14 of CCR5 lies near Arg285. Furthermore, the modeled positions of T16 and S17 are located near Arg106 and the Ala201 backbone, respectively. These observations suggest that the hTPST1 substrate‐binding pocket can accommodate multiple local sequence contexts through a combination of hydrophobic and electrostatic contacts.

For Y14 sulfation, Y15 may already be sulfated (sY15), whereas for Y15 sulfation, Y14 may already be sulfated (sY14). Therefore, we additionally prepared hTPST1‐PAP‐CCR5 peptide docking models with sY15 for Y14 sulfation and with sY14 for Y15 sulfation (Fig. 4E,F; Fig. S9E,F). In the hTPST1‐PAP‐CCR5 peptide docking model with sY15 for Y14 sulfation, the modeled position of sY15 places its sulfo group near Arg106, consistent with a possible electrostatic interaction (Fig. 4E; Fig. S9E). Likewise, in the hTPST1‐PAP‐CCR5 peptide docking model with sY14 for Y15 sulfation, the modeled position of sY14 places its sulfo group near Lys119(B) and Arg123(B), consistent with possible electrostatic interactions (Fig. 4F; Fig. S9F). hTPSTs employ a substrate‐binding pocket rich in hydrophobic and electrostatic features, including an arginine cluster. The substrate‐binding pocket of hTPST1 is therefore likely to facilitate sulfation of multiple tyrosine residues in the N‐terminal region of CCR5, as also suggested by previous studies of hTPST1 and tick TPST [27, 32].

Of the four potential glycosylation sites (S6, S7, T16, and S17), glycosylation at S6 can plausibly occur even prior to Y3 sulfation as discussed above. S7 is positioned distal to the hTPST1 substrate‐binding pocket for Y3 and Y10 sulfation and is therefore unlikely to disturb these sulfation events. T16 and S17 are considered minor glycosylation sites and typically accept shorter glycans [22]. In our sulfation models, T16 and S17 lie adjacent to the hTPST1 active site during the recognition of Y14 and Y15 (Fig. S8C,D). Unlike the Y3 site, where glycosylation appears noninhibitory, glycosylation at these distal positions could potentially interfere with sulfation at Y14 and/or Y15. Although the published example involves N‐glycosylation (mouse IgG2a), previous reports have shown that glycosylation has the potential to hinder tyrosine sulfation [37]. These observations are consistent with our proposal.

Based on the CCR5 Y3 peptide crystal structure, we constructed sulfation models for the CCR5 peptide for Y10, Y14, and Y15 sulfation. These models suggest how multiple hydrophobic and electrostatic features of the hTPST1 substrate‐binding pocket could accommodate sulfation at each of the four tyrosine residues and how adjacent O‐glycosylation sites might be tolerated.

### Docking models of full‐length hTPST1‐PAP‐CCR5

Although the hTPST1‐PAP‐CCR5 peptide crystal structure and the CCR5 peptide sulfation models provide valuable insights into local interactions, they do not capture the spatial arrangement and conformational flexibility of the full‐length CCR5 receptor in a membrane environment. Therefore, we built full‐length hTPST1‐PAP‐CCR5 docking models by integrating the crystallographic coordinates with AlphaFold3 [33]‐generated full‐length models. In developing the full‐length hTPST1‐PAP‐CCR5 models, we used three structural criteria to guide assembly of the final model. First, hTPSTs possess a single transmembrane (TM) domain in their N‐terminal region. Second, CCR5 contains seven TM helices, and its N terminus is linked to the extracellular loop by a conserved disulfide bond between C20 and C269 (Fig. S1). Third, hTPSTs must be able to access all four tyrosine residues (Y3, Y10, Y14, and Y15) of CCR5, particularly Y15, the most C‐terminal sulfated tyrosine residue in the CCR5 N‐terminal domain. Taken together, these considerations guided refinement of a preliminary template generated by AlphaFold3. Specifically, we used the standard version of AlphaFold3 to generate an initial template for the Y15 sulfation state. Because the current version of AlphaFold3 does not support user‐input constraints, no user‐defined constraints were applied during AlphaFold3 inference. Instead, an unconstrained AlphaFold3 model was used only as an initial template, and biologically motivated arrangements were introduced afterward during manual model selection and refinement. Next, we replaced part of the model with our hTPST1‐PAP‐CCR5 peptide crystal structure and performed local energy minimization in MOE to obtain a full‐length hTPST1‐PAP‐CCR5 docking model for Y3 sulfation with Y3 positioned at the acceptor site (Fig. S10A). Based on this full‐length hTPST1‐PAP‐CCR5 docking model for Y3 sulfation, full‐length hTPST1‐PAP‐CCR5 docking models for Y10, Y14, and Y15 sulfation were also constructed (Fig. S10B; Fig. S11A,B). Each full‐length hTPST1‐CCR5 docking model consists of an hTPST1 dimer and a CCR5 monomer positioned relative to a membrane‐mimetic scaffold approximately 40 Å thick and represented by 50 oleic acid molecules. Because this assembly was used only to provide a qualitative membrane reference frame and was not treated as an equilibrated lipid bilayer, membrane geometry and specific lipid contacts should not be interpreted at atomic detail. The mammalian Golgi lipid bilayer is reported to be approximately 40 Å thick [38], while the luminal thickness of the Golgi cisternae varies from ~100 to 1000 Å depending on position [39, 40, 41]; accordingly, we placed the full‐length models of hTPST1 and CCR5 on the same membrane plane. In these models, hTPST1 is arranged around the luminal N‐terminal region of CCR5 in a manner that could present the tyrosine residues to the active site. To assess the accessibility of Y3 and Y15, we measured the Cα–Cα distance between the acceptor tyrosine and C20, which forms the C20–C269 disulfide bond. In the full‐length hTPST1‐PAP‐CCR5 docking model for Y3 sulfation, the Cα–Cα distance between Y3 and C20 is 17.4 Å, which is consistent with the possibility that the CCR5 N‐terminal backbone could reach the hTPST1 active site (Fig. S10A). In the full‐length hTPST1‐PAP‐CCR5 docking model for Y15 sulfation, the Cα–Cα distance between Y15 and C20 is 16.8 Å, suggesting that accommodation of Y15 would likely require a more extended backbone conformation (Fig. S11B). These full‐length docking models therefore provide one plausible framework in which hTPST1 could engage the CCR5 N‐terminal region and potentially access all four tyrosine residues for sulfation. These full‐length arrangements are presented as hypothesis‐generating models that provide a structural framework for future biochemical and cellular validation.

We also assessed the possibility of dual sulfation on a single full‐length CCR5 monomer by an hTPST1 dimer from a structural perspective. In the crystal structure, the Cα–Cα distance between the two Y3 residues in the two CCR5 peptides bound to the hTPST1 dimer is 29.1 Å. On average, the Cα–Cα distance between adjacent residues along a polypeptide chain is approximately 3.8 Å [42]. Based on these values, simultaneous accommodation of two tyrosine residues in the two active sites would be expected to require a separation of approximately eight or more residues, although this estimate does not account for backbone curvature or conformational rearrangement. Therefore, within the CCR5 N‐terminal region, a pair such as Y3 and Y14/Y15 is not inconsistent with dual engagement by an hTPST1 dimer, whereas other tyrosine pairs would appear less favorable under the same simplified assumptions. Consistent with this interpretation, we generated full‐length hTPST1‐PAP‐CCR5 models for dual sulfation at Y3 and Y14 or Y15 (Fig. S12A,B). These models suggest one plausible arrangement that could enable dual sulfation at Y3 and Y14/Y15 and is qualitatively consistent with the reported sulfation order for the CCR5 Nt1–18 peptide by hTPST2 (first: Y3; second: Y14/Y15; last: Y10) [24].

hTPST1 is thought to form a homodimer mediated by both its transmembrane and catalytic domains [31, 43]. In the soluble domain, crystal structures of hTPST1 show a homodimer in which substrate‐recognition residues (e.g., Trp114 and Arg123) are provided by the partnering protomer [27]. In the TM domain, polar residues such as Cys13, Ser17, Ser18, and Thr20 have been proposed as candidate residues for dimer formation [43], but no experimentally determined TM interface residues have been reported. In our full‐length models, the shortest side‐chain–side‐chain distances between residues in opposing TM helices were approximately 15–20 Å, indicating that these polar residues are not engaged in interprotomer contacts (Fig. 5A). Nevertheless, this docking model represents one plausible arrangement under our modeling conditions and does not exclude alternative TM interfaces *in vivo*. While monomeric TPST variants retain some catalytic activity, dimerization may enhance substrate recognition and overall catalytic efficiency by expanding the substrate‐binding surface. One possible interpretation of the full‐length docking model is that a single CCR5 N‐terminal tail may be long enough to sample both active sites of the hTPST1 dimer, although this arrangement remains to be experimentally verified.

**Fig. 5:**
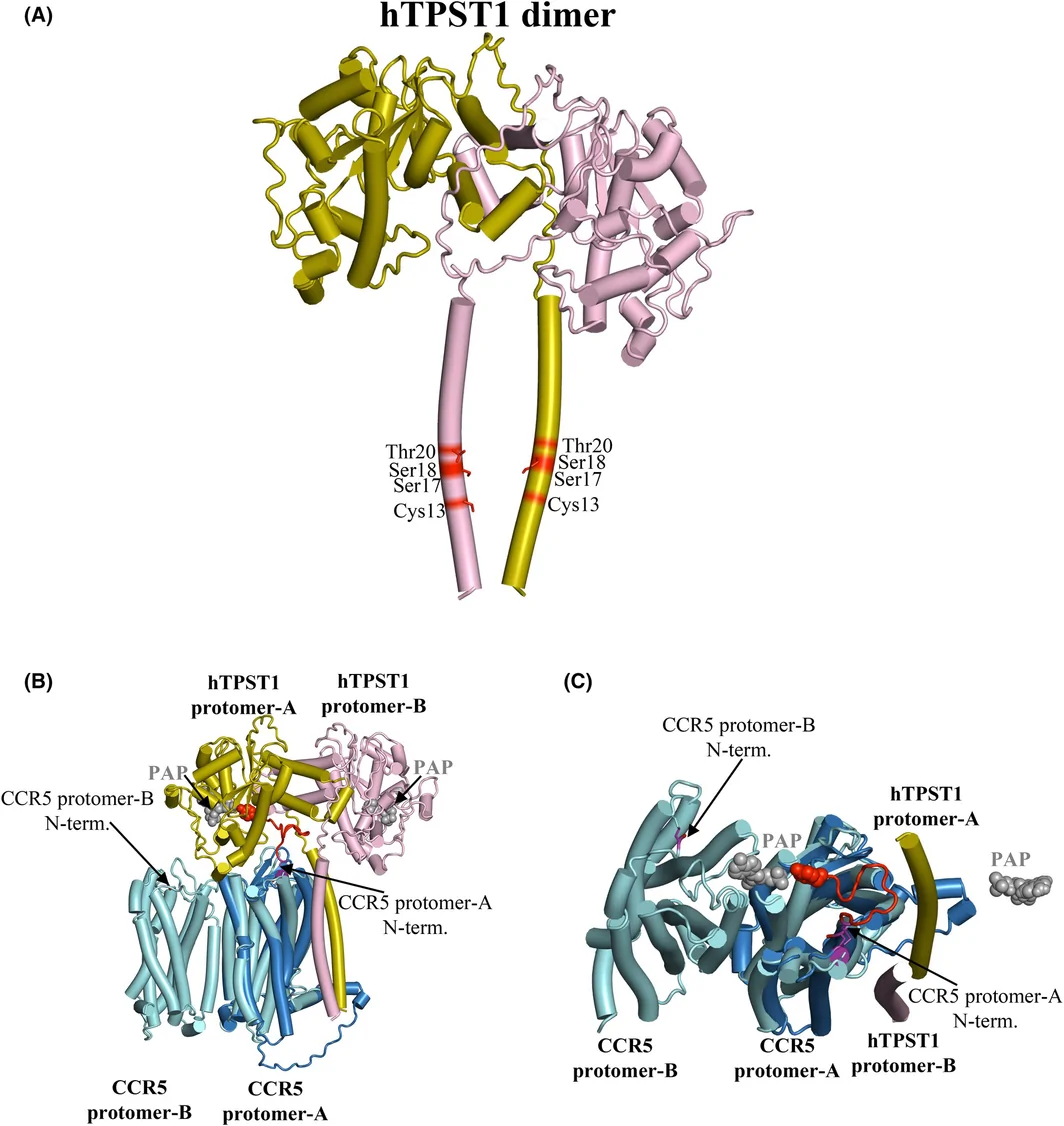
Full‐length hTPST1 dimer model and superposition of CCR5 TM domains between the full‐length hTPST1‐PAP‐CCR5 model and a CCR5 homodimer model. (A) Full‐length hTPST1 dimer, with candidate polar residues in the TM helices colored red. (B) Superposition of the full‐length hTPST1‐PAP‐CCR5 onto a CCR5 TM domain homodimer model. (C) Top view of panel (B). In panels (B) and (C), hTPST1 protomer‐A and protomer‐B are shown in olive and light pink, respectively. CCR5 monomer from the docking model is shown in sky blue and its N‐terminal region is highlighted in red. The CCR5 TM‐domain homodimer model is shown in cyan. PAP is shown as gray spheres, and the acceptor tyrosine residues are rendered as spheres.

Recent computational studies indicate that CCR5 can form TM4‐TM5‐mediated homodimers, and a CCR5 TM homodimer structural model is available [44]. Accordingly, we superposed a CCR5 TM homodimer model onto our full‐length hTPST1‐PAP‐CCR5 docking model to assess whether hTPSTs could sulfate tyrosine residues from both protomers of a CCR5 homodimer simultaneously. This superimposition places the N‐terminal residues of the second CCR5 protomer far from the hTPST1 active site (Fig. 5B,C), suggesting that simultaneous sulfation of both CCR5 protomers would appear sterically disfavored in this model unless substantial conformational rearrangements of hTPST1 occur. Experimental validation, such as mutagenesis analyses of the TM domain and additional simulations in explicit lipid bilayers, will be required to confirm the physiologically relevant arrangement.

In summary, the peptide‐based models together with the full‐length docking models support a working hypothesis in which hTPSTs could engage the luminal N‐terminal region of CCR5 and present its four N‐terminal tyrosine residues for sulfation. These putative models provide a conceptual framework for future analysis of other CCR family members that contain multiple sulfated tyrosine residues and a conserved disulfide bond in the N‐terminal region (Fig. S1). In this study, we used hTPST1 for crystal structure determination and construction of the docking models. Given the similarity of the available hTPST1 and hTPST2 crystal structures and substrate‐recognition modes [26, 27] and the reported ability of hTPST1 and hTPST2 to form heterodimers [31], some aspects of the present model may also be relevant to the hTPST2 homodimer and the hTPST1–hTPST2 heterodimer; however, this remains to be tested experimentally. The present crystal structure and the sulfation docking models provide structural insights into CCR sulfation and adjacent O‐glycosylation, with potential implications for viral and microbial infection and for chemokine signaling. These models may provide a structural framework for future studies aimed at modulating the sulfation status of CCRs. Future studies will undertake detailed analyses of the transmembrane domains of hTPSTs and their substrate proteins to gain a deeper understanding of the overall mechanism of tyrosine sulfation in the Golgi lumen.

## Conclusion

In conclusion, we determined the crystal structure of the hTPST1‐PAP‐CCR5 peptide complex and identified structural features consistent with CCR5 Y3 recognition/sulfation. Based on this structure, we generated sulfation docking models for CCR5 peptides containing Y10, Y14, and Y15, as well as full‐length hTPST1‐PAP‐CCR5 docking models for Y3, Y10, Y14, and Y15. Together, these findings provide structural insights into CCR5 sulfation with relevance to viral entry and immune responses. The full‐length arrangements are presented as hypothesis‐generating models that provide a structural framework for future biochemical and cellular validation.

## Materials and methods

### Preparation of recombinant hTPST1 and the CCR5 peptide

Recombinant soluble domain of hTPST1 (Lys43‐Glu370) was prepared using the same method as in the previous study [27]. The purified soluble domain of hTPST1 was stored in a buffer consisting of 50 mM Tris/HCl (pH 7.0) and 200 mM NaCl. The CCR5 peptide was chemically synthesized. The CCR5 peptide consisted of 18 amino acids (M1, D2, Y3, Q4, V5, S6, S7, P8, I9, E10, D11, I12, N13, E14, E15, T16, S17, E18), and Y10, Y14, and Y15 were substituted with glutamic acid to facilitate crystal structure determination with a single binding mode, as shown in Fig. 1B.

#### Enzymatic assay

The enzymatic assay was performed as described previously [27]. hTPST1 sulfotransferase activity was determined using radioactive [^35^S]PAPS. The reaction buffer contained 50 mM MES‐NaOH (pH 6.0), 20 mM MnCl_2_, 50 mM NaF, and 2 μg BSA. The reaction mixture also contained 0.5 μM [^35^S]PAPS as the donor substrate, the CCR5 peptide as the acceptor substrate, and 4.0 μg of the hTPST1 soluble domain. The sulfotransferase reaction was carried out for 15 min at 30 °C and quenched by heating at 100 °C for 3 min. The final reaction mixture was separated by thin‐layer chromatography using a solvent system of n‐butanol : pyridine : formic acid : water = 5 : 4 : 1 : 3. The TLC plate was dried, and the [^35^S]‐sulfated CCR5 peptide was quantified using a Fluoro Image Analyzer (Typhoon FLA9500). A representative TLC assay image showing the separation of [^35^S]PAPS and the sulfated CCR5 peptide is provided in Fig. S13.

#### Crystallization and data collection

Previous studies have shown that TPST substrate binding depends on the presence of PAPS or PAP and that PAP can act as a product inhibitor. In addition, the first hTPST2 structure revealed that PAP stabilizes the 5’‐PSB and 3’‐PB motifs [26]. Based on these observations, we reasoned that an hTPST1‐PAP‐CCR5 peptide complex would be sufficiently stable for crystallization, and we therefore performed co‐crystallization experiments with hTPST1, PAP, and the CCR5 peptide. We also note that the crystallization condition contained excess PAP (2 mM) and CCR5 peptide (1 mM), which may have further favored formation of the ternary complex in the crystal. Crystals of the hTPST1‐PAP‐CCR5 peptide complex were obtained using the sitting‐drop vapor‐diffusion method at 20 °C. While we also performed crystallization trials using modified CCR5 peptides centered on Y10, Y14, and Y15, these trials did not yield crystals suitable for diffraction analysis. Sitting drops were prepared by mixing 200 nL of sample solution with 200 nL of reservoir solution (1 : 1). The sample solution contained 5.0 mg·mL^−1^ hTPST1, 2 mM PAP, and 1 mM CCR5 peptide, whereas the reservoir solution contained 1.8 M sodium/potassium phosphate, pH 6.9. Crystals grew for approximately one month. For data collection, a crystal was transferred into cryo solution (1.8 M sodium/potassium phosphate [pH 6.9], 20% v/v glycerol) and then flash‐cooled in a cryo‐cooling system. X‐ray diffraction experiments were performed at beamline BL26B2 at SPring‐8 using a MarMosaic225 detector (Rayonix). The diffraction data were processed using HKL2000. The crystal belonged to space group *C2*, with unit‐cell parameters a = 188.9 Å, b = 189.4 Å, and c = 121.5 Å. Data collection statistics are summarized in Table 2.

#### Structural solution and refinement

The crystallographic phases were determined by molecular replacement using the hTPST2‐PAP‐C4 peptide (PDB ID: 3AP1) as the search model with Molrep [45]. The crystal structure was determined through iterative cycles of model building and refinement using Coot [34] and Phenix [46]. During refinement, weak electron density was observed in the C‐terminal region (V5 and S6). To ensure the correct chain tracing, we compared candidate models using Polder omit maps and Ramachandran plot analysis. The final refined model was selected because it placed V5 in the favored region, whereas the alternative swapped model resulted in poor geometry (Fig. S7A,B). Refinement statistics are summarized in Table 2. The crystal structure quality was further assessed by MolProbity [47], which indicated good overall geometries. The atomic coordinates and structure factors have been deposited in the Protein Data Bank under accession code 9WP1.

#### Construction of glycosylation models of the CCR5 peptide

The glycosylation models of the CCR5 peptide were constructed based on the hTPST1‐PAP‐CCR5 peptide crystal structure. S6 was identified as a potential glycosylation site. For the short glycan model, GalNAc was attached to S6, whereas for the long glycan model, a trisaccharide composed of GalNAc‐Gal‐Neu5Ac (Neu5Ac(α2–3)Gal(β1–3)GalNAc) was attached. The hydroxyl group of the S6 side chain and the attached glycans were energy‐minimized. These models comprised a Y3 sulfation model bearing S6‐GalNAc as a short glycan and a Y3 sulfation model bearing S6‐GalNAc‐Gal‐Neu5Ac as a long glycan.

#### Construction of hTPST1‐PAP‐CCR5 peptide sulfation models

We generated hTPST1‐PAP‐CCR5 peptide docking models for Y10, Y14, and Y15 sulfation using the crystal structures of the hTPST1‐PAP‐C4 peptide, hTPST1‐PAP‐gastrin peptide, and hTPST1‐PAP‐CCR5 peptide. Since the overall fold and catalytic residues are conserved among the three distinct hTPST1 structures, we energy‐minimized only the CCR5 peptide residues in the crystal structure of the hTPST1‐PAP‐CCR5 peptide using MOE (2025; Chemical Computing Group Inc.). hTPST1‐PAP‐CCR5 peptide docking models for Y10, Y14, and Y15 sulfation were constructed based on the energy‐minimized hTPST1‐PAP‐CCR5 Y3 peptide crystal structure. First, the CCR5 peptide in the hTPST1‐PAP‐CCR5 peptide crystal structure was energy‐minimized in MOE, and residues other than the acceptor Y were mutated to generate the Y10, Y14, and Y15 peptide sequences, respectively. The resulting peptides were then energy‐minimized. This procedure yielded hTPST1‐PAP‐CCR5 peptide docking models for Y10, Y14, and Y15 sulfation. For the hTPST1‐PAP‐CCR5 docking model with sY15 for Y14 sulfation and the hTPST1‐PAP‐CCR5 peptide docking model with sY14 for Y15 sulfation, sulfo groups were added to the hydroxyl groups of Y15 or Y14 in the respective sulfation models, and each peptide was energy‐minimized. For the hTPST1‐PAP‐CCR5 sY14Y15 peptide model, Lys119(B) lies within interaction distance of sY14; therefore, its side chain was energy‐minimized.

#### Construction of full‐length hTPST1‐PAP‐CCR5 docking models

The docking models of the full‐length hTPST1 dimer and full‐length CCR5 were established based on the hTPST1‐PAP‐CCR5 peptide crystal structure. To produce a preliminary template, the sequences of the hTPST1 dimer and full‐length CCR5, together with 50 oleic acid molecules as lipid‐bilayer mimetics, were input into the publicly accessible AlphaFold3 server [33]. No custom template restraints or user‐defined structural constraints were applied during AlphaFold3 inference. The oleic acid assembly was used only as a membrane‐mimetic scaffold to place the transmembrane segments in a common reference frame and was not treated as an equilibrated lipid bilayer; therefore, membrane geometry and specific lipid contacts should be interpreted qualitatively. To facilitate positioning of the N‐terminal tail, Y3, Y10, and Y14 of CCR5 were mutated to alanine residues in the input sequence, leaving Y15 as the most C‐terminal candidate sulfation site. After AlphaFold3 prediction, the catalytic domain and bound peptide region were replaced with the experimentally determined crystal structure coordinates for hTPST1 (Ala64‐Asp339) and CCR5 (M1–S6). The remaining N‐ and C‐terminal regions of hTPST1 and the CCR5 extracellular segment (S7–P19) were then manually adjusted in Coot and refined by local energy minimization in MOE to satisfy the structural criteria described in the Results and Discussion section. The resulting model was used as the full‐length hTPST1‐PAP‐CCR5 docking model for Y3 sulfation. Subsequently, Y3 was replaced *in silico* with Y10, Y14, or Y15 as the acceptor residue, and the surrounding residues were mutated accordingly, followed by manual adjustment in Coot and local energy minimization in MOE to generate the full‐length hTPST1‐PAP‐CCR5 docking models for Y10, Y14, and Y15 sulfation.

To construct the full‐length hTPST1‐PAP‐CCR5 model for dual sulfation at Y3 and Y14, the full‐length hTPST1‐PAP‐CCR5 model for Y14 sulfation was used as a template. The opposite protomer of hTPST1 in the Y14 sulfation model was superposed with protomer‐A of the hTPST1‐PAP‐CCR5 peptide crystal structure (hTPST1 Ala64‐Asp339 and CCR5 M1–S6), and CCR5 M1–S6 was replaced for dual sulfation for Y3 and Y14. The region spanning S7–N13 between the crystallographic segment (M1–S6) and Y14 was then adjusted in Coot and energy‐minimized in MOE. This procedure yielded the full‐length hTPST1‐PAP‐CCR5 docking model for dual sulfation at Y3 and Y14.

To construct the full‐length hTPST1‐PAP‐CCR5 model for dual sulfation at Y3 and Y15, the full‐length hTPST1‐PAP‐CCR5 model for Y15 sulfation was used as a template. The opposite protomer of hTPST1 in the Y15 sulfation model was superposed with protomer‐A of the hTPST1‐PAP‐CCR5 peptide crystal structure (hTPST1 Ala64‐Asp339 and CCR5 M1–S6), and CCR5 M1‐S6 was replaced for dual sulfation for Y3 and Y15. The region spanning S7–Y14 between the crystallographic segment (M1–S6) and Y15 was then adjusted in Coot and energy‐minimized in MOE. This procedure yielded the full‐length hTPST1‐PAP‐CCR5 docking model for dual sulfation at Y3 and Y15.

## Author contributions

ST, HA, KT, TN, HK, EN, TT, and YK performed experiments (protein production, crystallographic analysis, and molecular modeling) and analyzed data. YS, KKu, and KKi performed the enzymatic assay. ST and YK wrote the paper. YK devised the project.

## Conflict of interest

The authors declare no conflict of interest.

## Supporting information


**Fig. S1.** Multiple‐sequence alignment of human chemokine receptors (CCR1–10).
**Fig. S2.** Amino acid sequences of the complement C4 peptide (C4P5Y5 peptide) and the gastrin peptide.
**Fig. S3.** Superposition of dimers and protomers in the asymmetric unit.
**Fig. S4.** Superposition of hTPST1 protomer‐A from hTPST1‐PAP‐C4 peptide, hTPST1‐PAP‐gastrin peptide, and hTPST1‐PAP‐CCR5 peptide.
**Fig. S5.** Substrate binding pocket of hTPST1‐PAP‐CCR5 peptide and hTPST2‐PAP‐CCR5 peptide.
**Fig. S6.** Interactions between hTPST1 and C4 peptide in the crystal structure.
**Fig. S7.** Polder omit maps support the final assignment of the CCR5 C‐terminal residues (V5 and S6).
**Fig. S8.** Glycosylation‐related views of the CCR5 peptide in the hTPST1‐PAP‐CCR5 models.
**Fig. S9.** Surface models of hTPST1‐PAP‐CCR5 peptide and hTPST1‐PAP‐CCR5 Y10, Y14, and Y15 peptide docking models.
**Fig. S10.** Full‐length hTPST1‐PAP‐CCR5 docking models for Y3 and Y10 sulfation.
**Fig. S11.** Full‐length hTPST1‐PAP‐CCR5 docking models for Y14 and Y15 sulfation.
**Fig. S12.** Full‐length hTPST1‐PAP‐CCR5 docking models for Y3 and Y14/Y15 dual sulfation.
**Fig. S13.** Representative TLC analysis of CCR5 peptide sulfation.

## Data Availability

Atomic coordinates and structure factors for the hTPST1‐PAP‐CCR5 peptide complex centered on Y3 have been deposited in the Protein Data Bank under accession code 9WP1. All docking and glycosylation‐related models described in this study have been deposited in ModelArchive. The hTPST1‐PAP‐CCR5 peptide docking and glycosylation‐related models for Y10, Y14, and Y15 sulfation shown in Fig. 4B–F, Fig. S8C,D, and Fig. S9B–F are available from ModelArchive as follows. They were prepared for CCR5 peptide bound to protomer‐A of hTPST1. ma‐9qbbb Human tyrosylprotein sulfotransferase 1–CCR5 (Y10 peptide) Structure‐Guided Docking Models (Template: PDB 9WP1) DOI: 10.5452/ma‐9qbbb. ma‐87ygk Human tyrosylprotein sulfotransferase 1–CCR5 (Y14 peptide) Structure‐Guided Docking Models (Template: PDB 9WP1) DOI: 10.5452/ma‐87ygk. ma‐wpmx3 Human tyrosylprotein sulfotransferase 1–CCR5 (Y15 peptide) Structure‐Guided Docking Models (Template: PDB 9WP1) DOI: 10.5452/ma‐wpmx3. ma‐81tbc Human tyrosylprotein sulfotransferase 1–CCR5 (Y14‐sY15 peptide) Structure‐Guided Docking Models (Template: PDB 9WP1) DOI: 10.5452/ma‐81tbc. ma‐cdi1r Human tyrosylprotein sulfotransferase 1–CCR5 (sY14‐Y15 peptide) Structure‐Guided Docking Models (Template: PDB 9WP1) DOI: 10.5452/ma‐cdi1r. The glycosylation models of the CCR5 peptide shown in Fig. S8A,B are available from ModelArchive as follows. They were prepared for CCR5 peptide bound to protomer‐A of hTPST1. ma‐6an7y Human tyrosylprotein sulfotransferase 1–CCR5 (Y3‐S6‐GalNAc peptide) Structure‐Guided Docking Models (Template: PDB 9WP1) DOI: 10.5452/ma‐6an7y. ma‐7g22u Human tyrosylprotein sulfotransferase 1–CCR5 (Y3‐S6‐GalNAc‐Gal‐Neu5Ac peptide) Structure‐Guided Docking Models (Template: PDB 9WP1) DOI: 10.5452/ma‐7g22u. The docking models of full‐length hTPST1‐PAP‐CCR5 shown in Figs S10A,B, S11A,B, S12A,B, and 5A–C are available from ModelArchive as follows: ma‐3bvoc Human tyrosylprotein sulfotransferase 1–CCR5 full length (Y3) Structure‐Guided Docking Models (Template: PDB 9WP1) DOI: 10.5452/ma‐3bvoc. ma‐ti3gu Human tyrosylprotein sulfotransferase 1–CCR5 full length (Y10) Structure‐Guided Docking Models (Template: PDB 9WP1) DOI: 10.5452/ma‐ti3gu. ma‐lsyav Human tyrosylprotein sulfotransferase 1–CCR5 full length (Y14) Structure‐Guided Docking Models (Template: PDB 9WP1) DOI: 10.5452/ma‐lsyav. ma‐k9kwx Human tyrosylprotein sulfotransferase 1–CCR5 full length (Y15) Structure‐Guided Docking Models (Template: PDB 9WP1) DOI: 10.5452/ma‐k9kwx. ma‐90 rpm Human tyrosylprotein sulfotransferase 1–CCR5 full length (Y3, Y14) structure‐guided docking model (template: PDB 9WP1). DOI: 10.5452/ma‐90 rpm. ma‐ukhc9 Human tyrosylprotein sulfotransferase 1–CCR5 full length (Y3, Y15) structure‐guided docking model (template: PDB 9WP1). DOI: 10.5452/ma‐ukhc9.
