# Supplementary material for: Structural insights into tyrosine sulfation of CCR5 by human tyrosylprotein sulfotransferase‐1
Source: FEBS J. 2026 May 22;293(21):6552–68. doi: 10.1111/febs.70597 (PMC13731920; doi:10.1111/febs.70597)
Supplement: Supplementary file 1 [file FEBS-293-6552-s001.pdf]

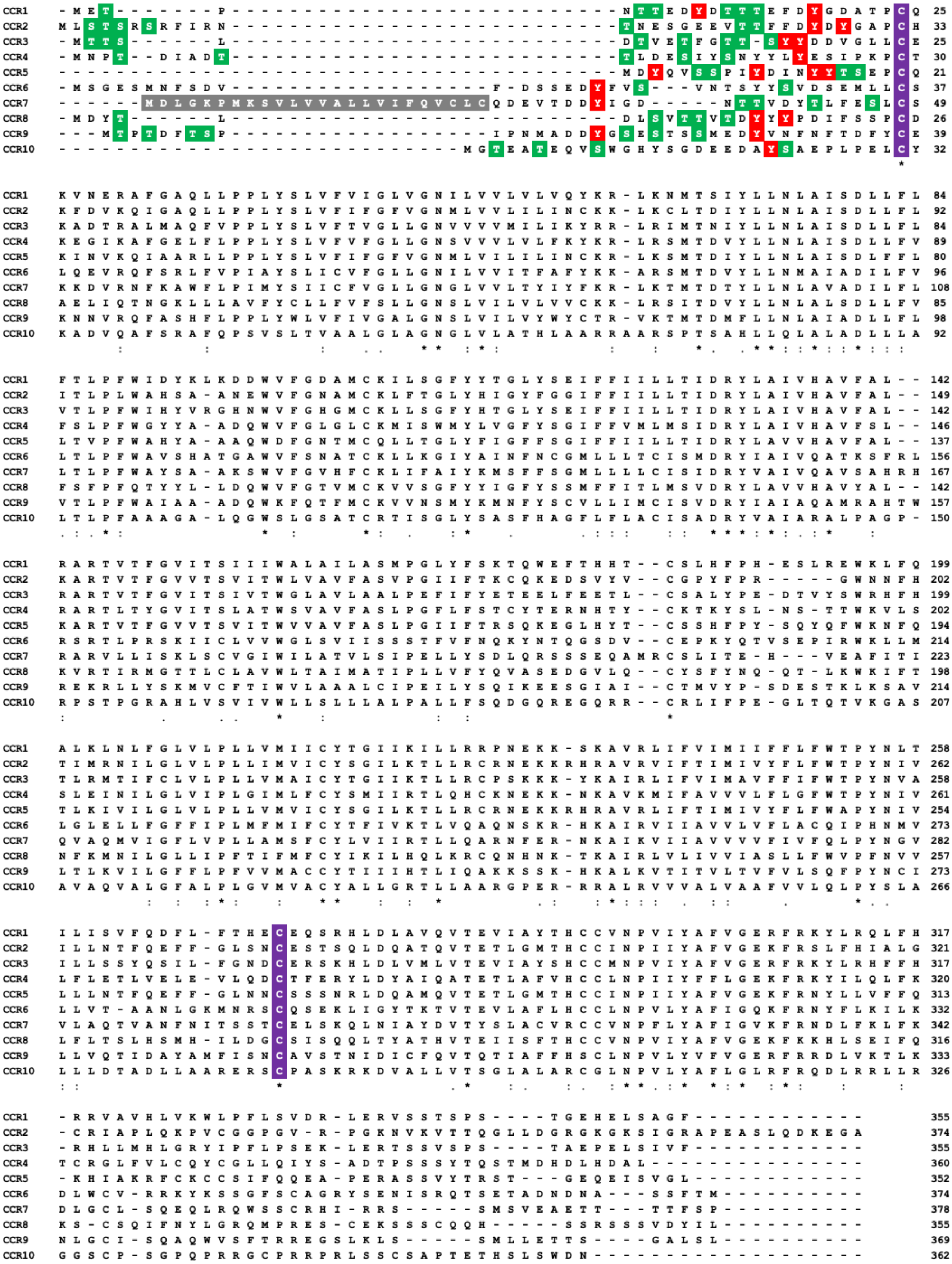

Figure S1. Multiple-sequence alignment of human chemokine receptors (CCR1–10).
Canonical amino acid sequences of human CCR1–CCR10 were retrieved from UniProtKB [CCR1 (P32246), CCR2 (P41597-1), CCR3 (P51677-1), CCR4 (P51679), CCR5 (P51681), CCR6 (P51684), CCR7 (P32248), CCR8 (P51685-1), CCR9 (P51686-1), and CCR10 (P46092)] and aligned using Clustal Omega [13].

Figure S1. Continued.
The tyrosine sulfation sites and conserved cysteine residues of human chemokine receptors (CCR1–10) are depicted. Predicted tyrosine sulfation sites and O-glycosylation sites are shown in red and green, respectively [15]. Conserved cysteine residues involved in the disulfide bond in the N-terminal domain are shown in purple. The CCR7 N-terminus region includes a unique signal sequence and is shown in gray.

**complement C4 WT**		…	E^{736}	D^{737}	Y^{738}	E^{739}	D^{740}	Y^{741}	E^{742}	Y^{743}	D^{744}	E^{745}	L^{746}	…
**complement C4 peptide**			E^{-5}	D^{-4}	F^{-3}	E^{-2}	D^{-1}	$Y^{acceptor}$	E^{+1}	F^{+2}	D^{+3}	E^{+4}	L^{+5}	
**gastrin WT**	…	E^{81}	E^{82}	E^{83}	E^{84}	E^{85}	A^{86}	Y^{87}	G^{88}	W^{89}	M^{90}	D^{91}	F^{92}	…
**gastrin peptide**		E^{-6}	E^{-5}	E^{-4}	E^{-3}	E^{-2}	A^{-1}	$Y^{acceptor}$	G^{+1}	W^{+2}	M^{+3}	D^{+4}	F^{+5}	

Red: Tyrosine sulfation site

Gray: Disordered region in productive complex

Figure S2. Amino acid sequences of the complement C4 peptide (C4P5Y5 peptide) and the gastrin peptide.

The wild-type sequences of the complement C4 and gastrin peptides are shown on upper row, with amino acid sequence numbers and subsite numbers indicated as superscripts on lower row, according to the previous report [27]. Y^{738} and Y^{743} in the C4 peptide were mutated to phenylalanine for crystallographic analysis.

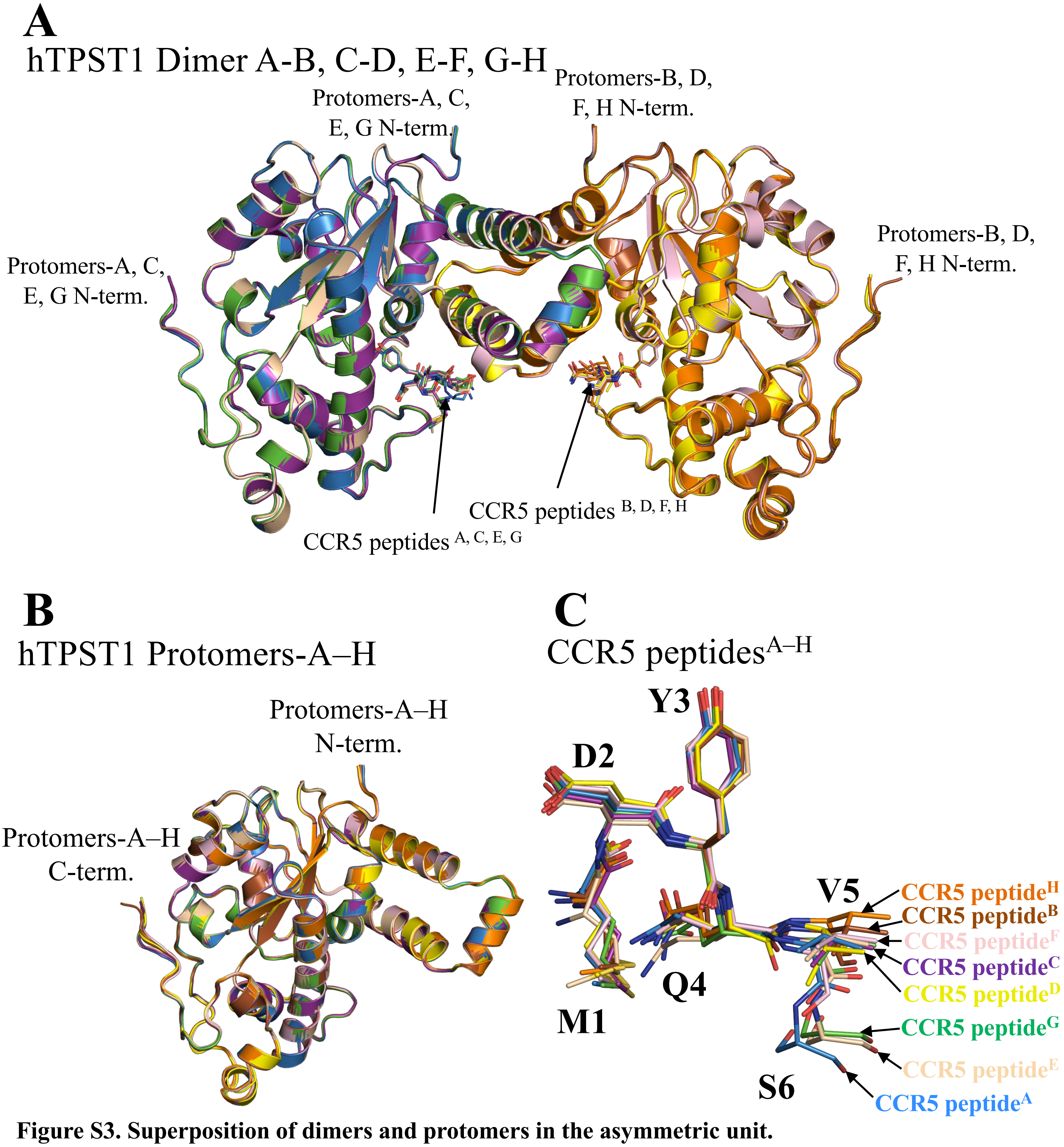

Figure S3. Superposition of dimers and protomers in the asymmetric unit.

(A) Cartoon representation of dimer C-D, dimer E-F, and dimer G-H superposed onto dimer A-B with CCR5 peptides$^{A\text{–}H}$ shown as sticks. In the asymmetric unit, protomers A–B, C–D, E–F, and G–H form dimer pairs. Pairwise superposition of dimer A/B with dimers C/D, E/F, and G/H yielded RMSDs of 0.31 Å over 552 atoms, 0.24 Å over 551 atoms, and 0.34 Å over 551 atoms, respectively. (B) Cartoon representations of protomers-A–H superposed onto protomer-A. (C) Stick representations of CCR5 peptidesA–H shown in the same superposition as in (B). Here, the CCR5 peptides in protomers-A–H are referred to as CCR5 peptideA–H. Protomers-A–H are colored sky blue, brown, purple, yellow, wheat, light pink, green, and orange, respectively. In the asymmetric unit, protomers A–B, C–D, E–F, and G–H form dimer pairs.

Figure S3. Continued.
The structural models shown in panels A–C are based on the X-ray crystal structure of the hTPST1-PAP-CCR5 peptide complex determined in this study. Structural superpositions were performed using Coot [45], and molecular graphics were prepared using PyMOL.

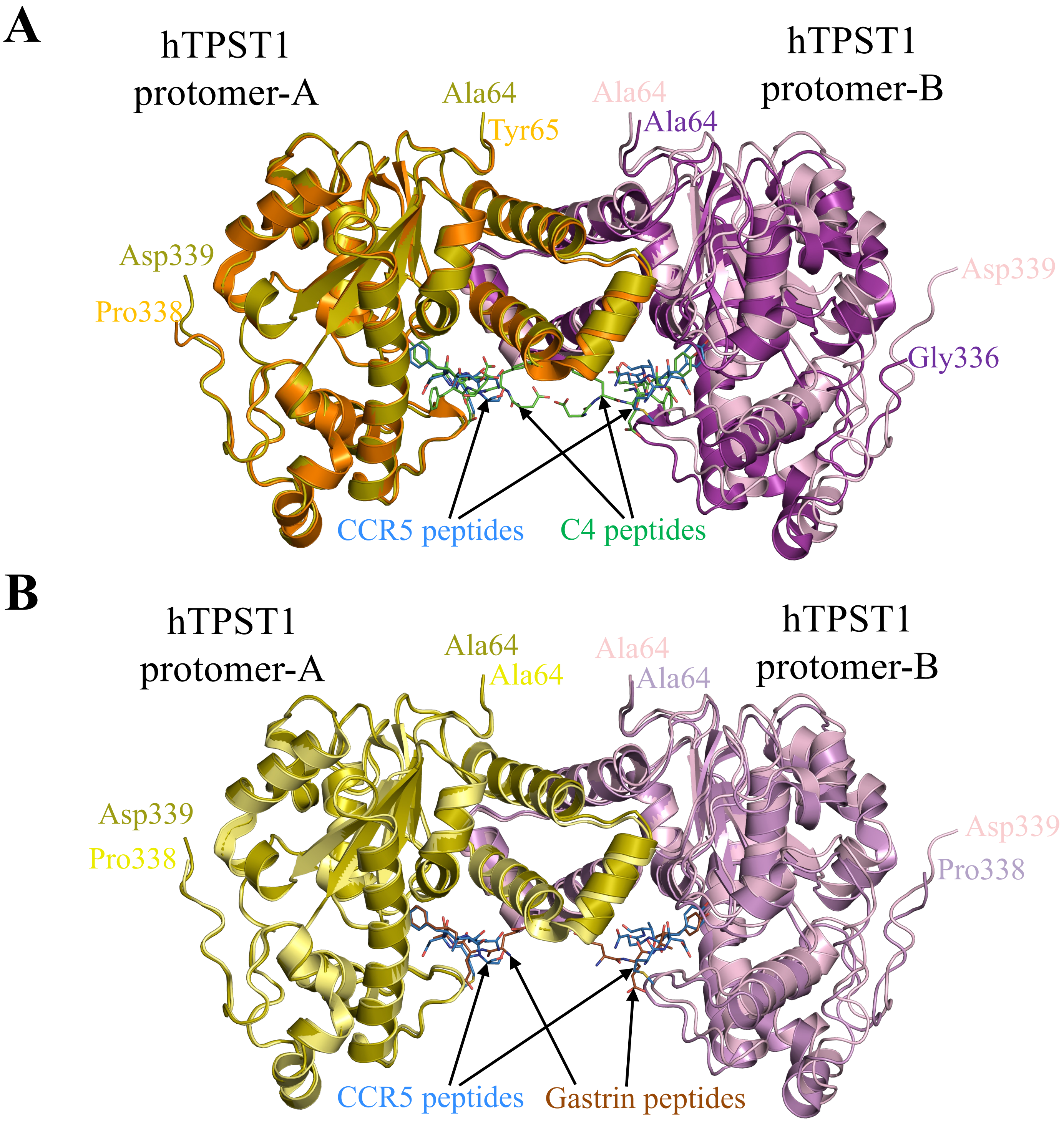

Figure S4. Superposition of hTPST1 protomer-A from hTPST1-PAP-C4 peptide, hTPST1-PAP-gastrin peptide, and hTPST1-PAP-CCR5 peptide.

(A) Superposition of protomer-A from the hTPST1-PAP-CCR5 peptide and hTPST1-PAP-C4 peptide. Protomer-A is colored olive (Ala64–Asp339) for the CCR5 peptide complex and orange (Tyr65–Pro338) for the C4 peptide complex, respectively. Protomer-B is colored light pink (Ala64–Asp339) for the CCR5 peptide complex and purple (Ala64–Gly336) for the C4 peptide complex, respectively. The CCR5 peptide and the C4 peptide are colored in sky blue and green, respectively. (B) Superposition of protomer-A from the hTPST1-PAP-CCR5 and the hTPST1-PAP-gastrin peptide. Protomer-A is colored olive (Ala64–Asp339) for the CCR5 peptide complex and pale yellow (Ala65–Pro338) for the gastrin peptide complex, respectively. Protomer-B is colored light pink (Ala64–Asp339) for the CCR5 peptide complex and violet (Ala64–Pro338) for the gastrin peptide complex, respectively. The CCR5 peptide and the gastrin peptide are colored in sky blue and brown, respectively.

Figure S4. Continued.

The structural models shown in panels A and B are based on the X-ray crystal structure of the hTPST1-PAP-CCR5 peptide complex determined in this study and the previously reported X-ray crystal structures of the hTPST1-PAP-C4 peptide and hTPST1-PAP-gastrin peptide complexes [27]. Structural superpositions were performed, and molecular graphics were prepared, using PyMOL.

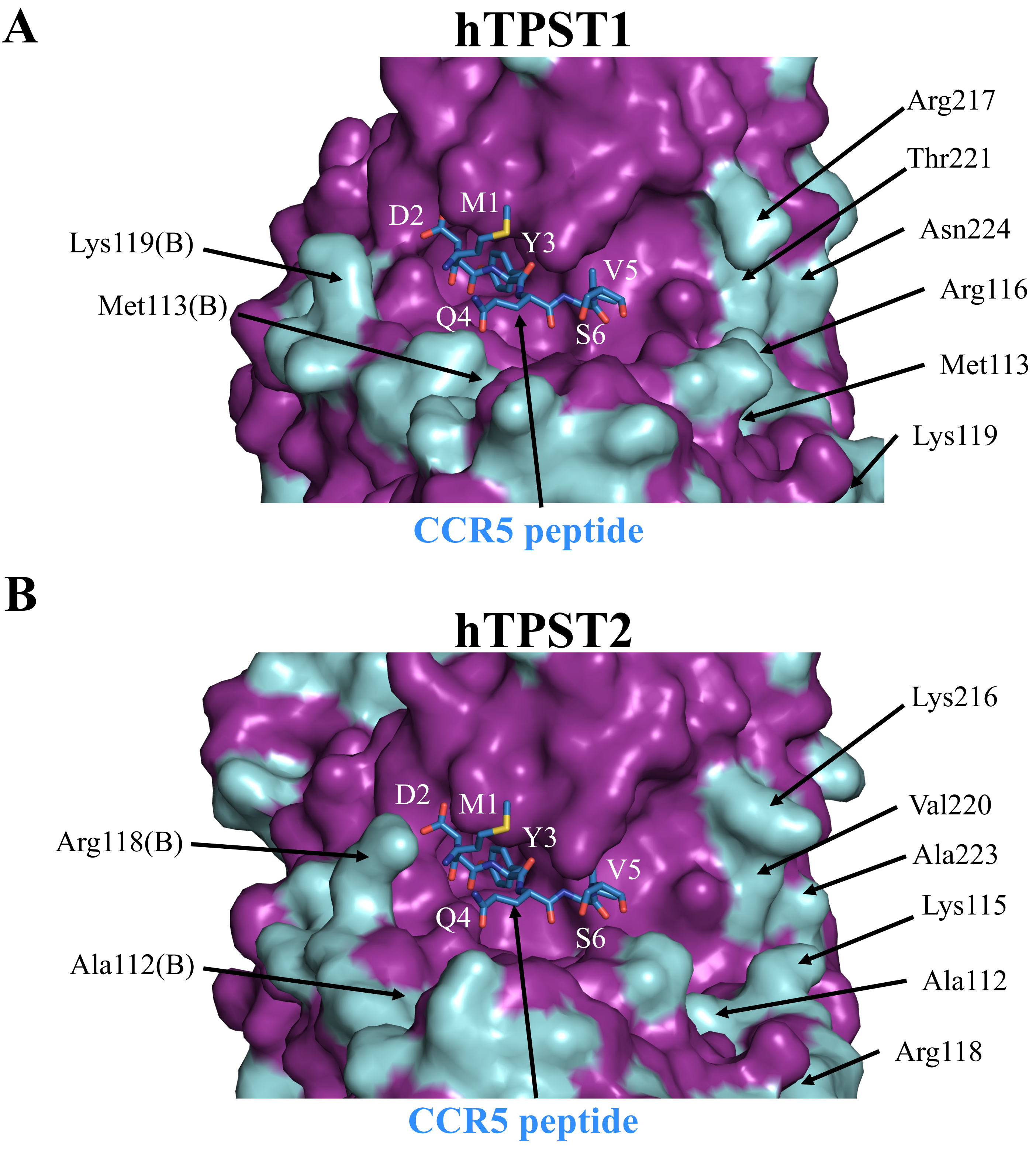

Figure S5. Substrate binding pocket of hTPST1-PAP-CCR5 peptide and hTPST2-PAP-CCR5 peptide. (A) Surface representation of hTPST1-PAP-CCR5 peptide. (B) Surface representation of hTPST2-PAP-CCR5 peptide. hTPST2-PAP-CCR5 peptide model was generated by superposing the hTPST2-PAP-C4 peptide structure onto the hTPST1-PAP-CCR5 peptide structure and replacing the C4 peptide with the CCR5 peptide. In (A) and (B), residues identical between hTPST1 and hTPST2 are shown in purple, and non-identical residues are shown in cyan. The CCR5 peptide is shown as sky-blue sticks.

Figure S5. Continued.

The structural model shown in panel A is based on the X-ray crystal structure of the hTPST1-PAP-CCR5 peptide complex determined in this study. The model shown in panel B is based on the previously reported X-ray crystal structure of the hTPST2-PAP-C4 peptide complex [26] and was generated by structural superposition with the hTPST1-PAP-CCR5 peptide complex, followed by replacement of the C4 peptide with the CCR5 peptide. No additional modeling or energy minimization was performed. Structural superpositions were performed using Coot [45], and molecular graphics were prepared using PyMOL.

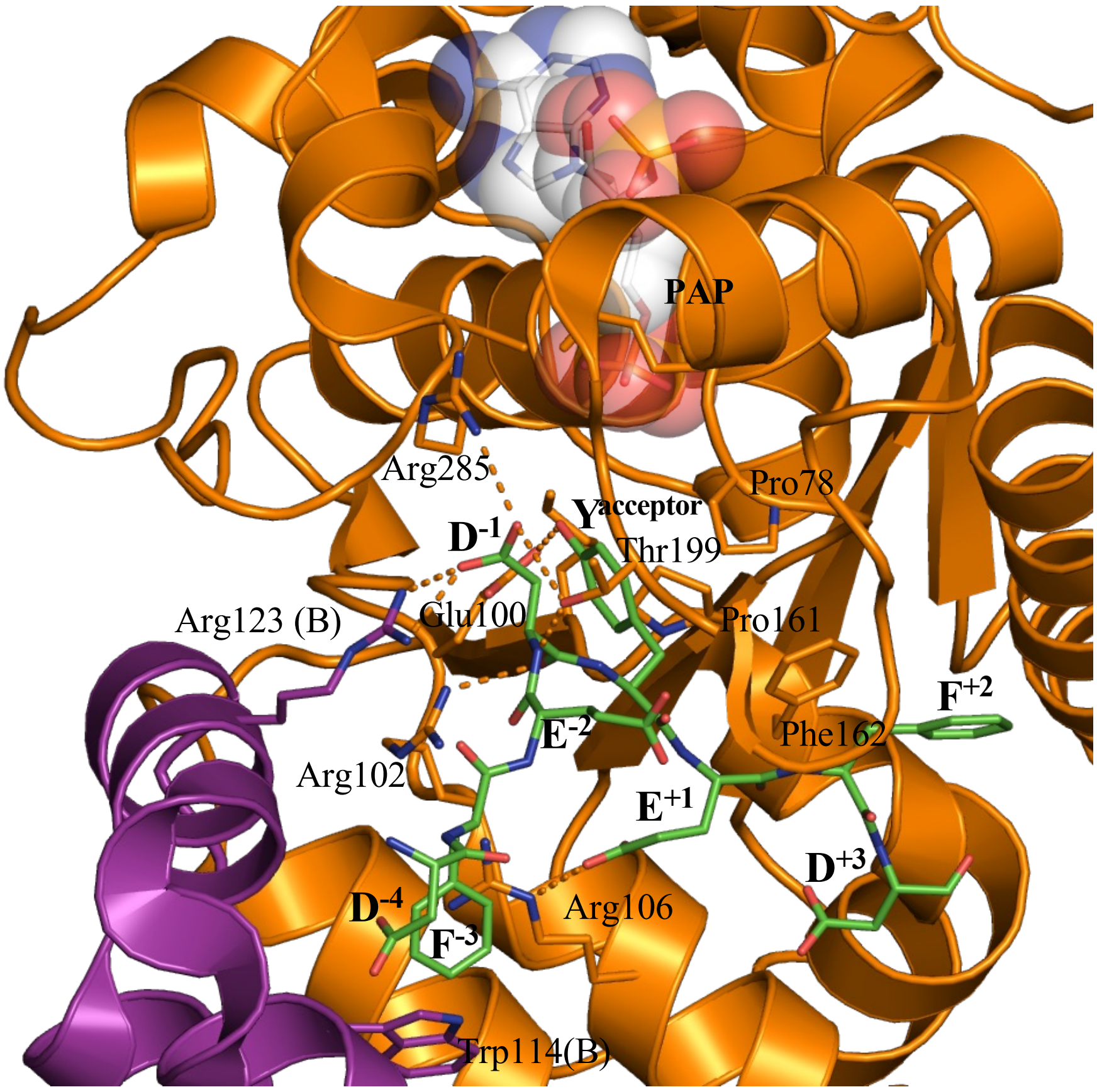

Figure S6. Interactions between hTPST1 and C4 peptide in the crystal structure.
Protomer-A and protomer-B of hTPST1 are shown in orange and purple ribbon models, respectively. The C4 peptide is shown as a green stick model, and the hydrogen bonds to hTPST1 are dashed orange lines. PAP is shown as a stick model and as a semi-transparent sphere model. The structural model is based on the previously reported X-ray crystal structure of the hTPST1-PAP-C4 peptide complex [27]. Molecular graphics were prepared using PyMOL.

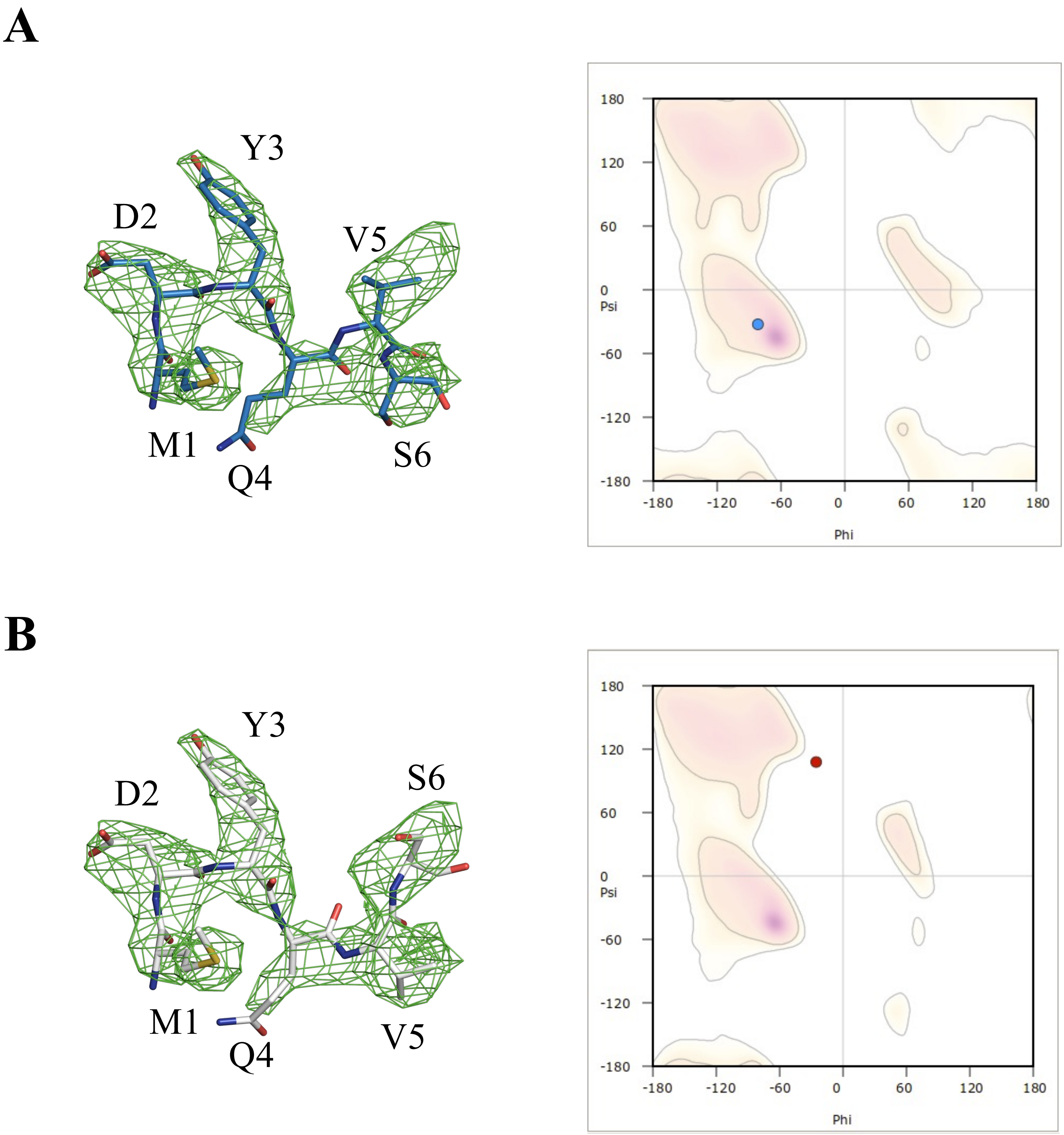

Figure S7. Polder omit maps support the final assignment of the CCR5 C-terminal residues (V5 and S6).

(A) Left: Polder omit map (green mesh) with the final refined model (sky blue stick) for the CCR5 C-terminal segment. Right: Ramachandran plot showing the backbone conformation of V5 (blue dot) in the favored region. (B) Left: Polder omit map with an alternative model in which the positions of V5 and S6 are swapped (inverted tracing). Right: Ramachandran plot for V5 in the swapped model (red dot), which falls in an outlier region, indicating that the swapped tracing is not supported.

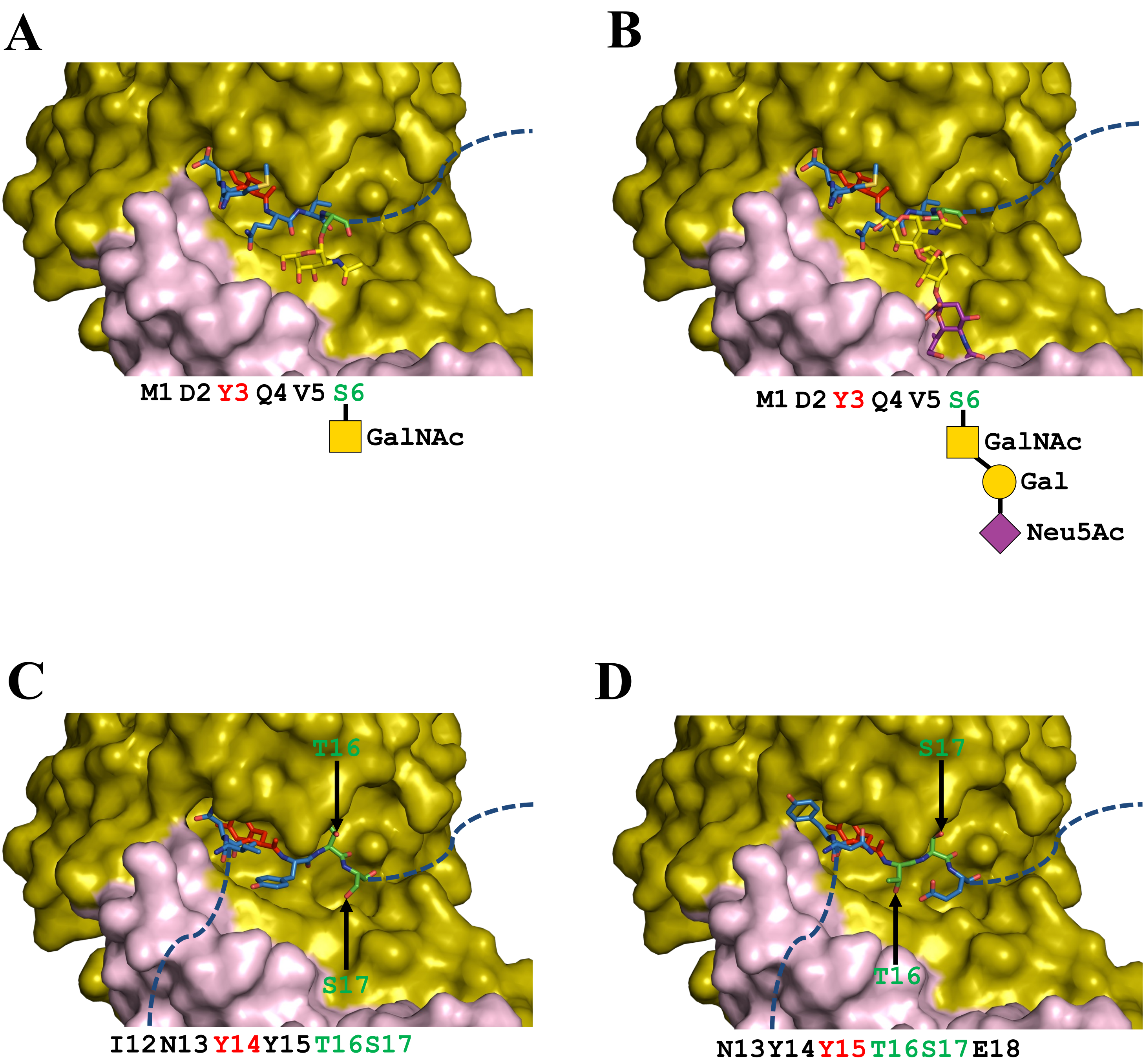

Figure S8. Glycosylation-related views of the CCR5 peptide in the hTPST1-PAP-CCR5 models. (A) Y3 sulfation model with S6-GalNAc. (B) Y3 sulfation model with S6-GalNAc-Gal-Neu5Ac. (C) Y14 sulfation docking model highlighting T16 and S17 as potential O-glycosylation sites. (D) Y15 sulfation docking model highlighting T16 and S17 as potential O-glycosylation sites. In the (C) and (D), black arrows indicate the hydroxyl groups of T16 and S17 (potential O-glycosylation sites). hTPST1 protomer-A and protomer-B are displayed as surface models in olive and light pink, respectively. Glycan symbols: GalNAc (yellow square), galactose (yellow circle), Neu5Ac (purple diamond). Potential O-glycosylation sites (S6, T16, and S17) are shown in green, and the CCR5 peptide is shown in sky blue. GalNAc and galactose are rendered in yellow, and Neu5Ac is shown in purple. Dashed lines show the modeled N-terminal main chain and C-terminal main chains.

Figure S8. Continued.

The structural models shown in panels A and B are based on the X-ray crystal structure of the hTPST1-PAP-CCR5 peptide complex determined in this study, and the glycan moieties were modeled and energy-minimized using the Molecular Operating Environment (MOE; Chemical Computing Group). The hTPST1 structures shown in panels C and D are based on the X-ray crystal structure determined in this study, and the CCR5 peptides were modeled and energy-minimized using MOE. Molecular graphics were prepared using PyMOL.

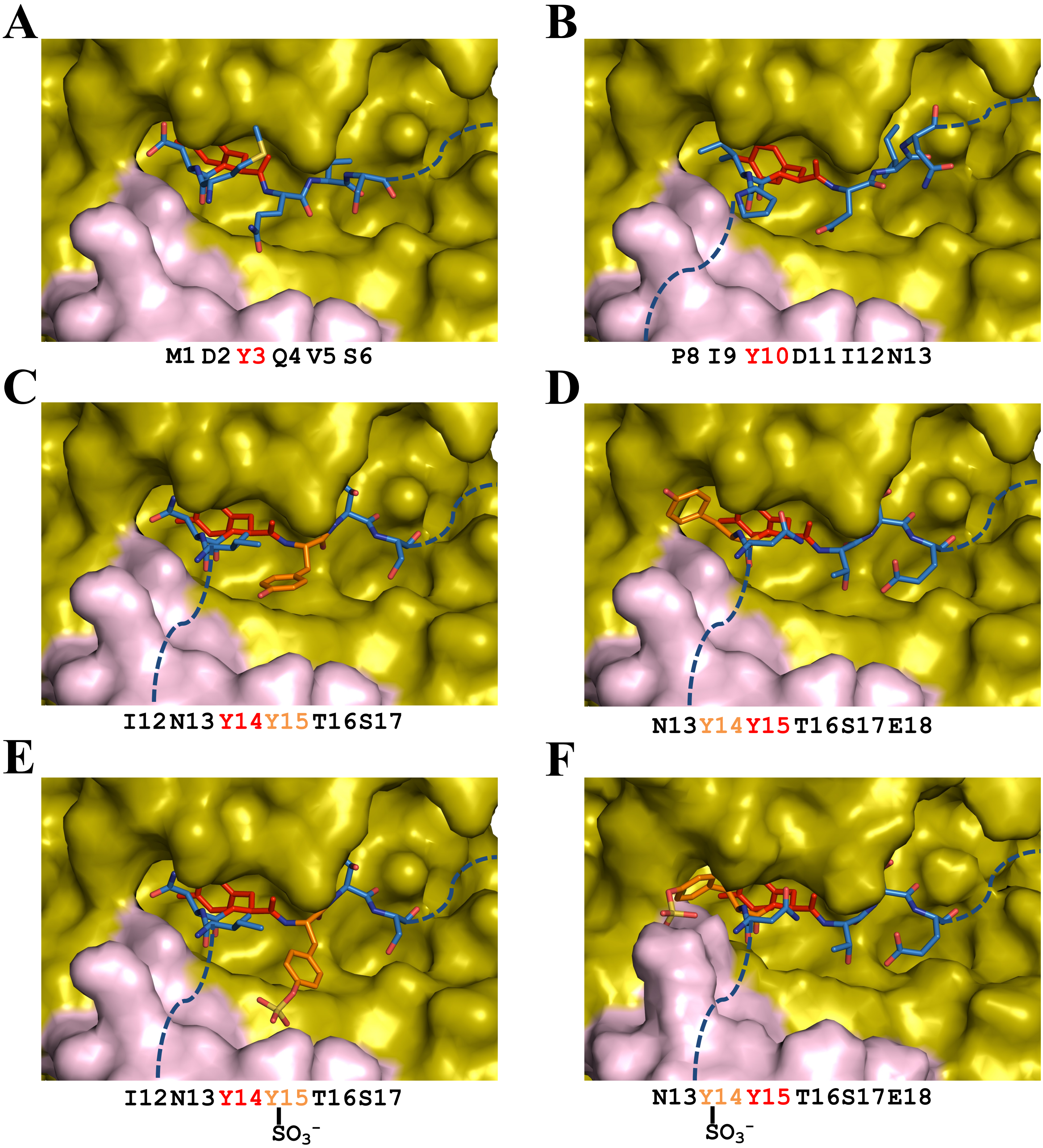

Figure S9. Surface models of hTPST1-PAP-CCR5 peptide and hTPST1-PAP-CCR5 Y10, Y14, and Y15 peptide docking models.

Protomer-A and protomer-B of hTPST1 are shown as surface models in olive and light pink, respectively. Each CCR5 peptide is rendered in sky blue, with acceptor tyrosine residues highlighted in red. Non-acceptor (other) tyrosine residues are rendered in orange. Dashed lines indicate modeled N-terminal and C-terminal main chains. (A) hTPST1-PAP-CCR5 peptide docking model for Y3 sulfation (crystal structure). (B) hTPST1-PAP-CCR5 peptide docking model for Y10 sulfation. (C) hTPST1-PAP-CCR5 peptide docking model for Y14 sulfation. (D) hTPST1-PAP-CCR5 peptide docking model for Y15 sulfation. (E) hTPST1-PAP-CCR5 peptide docking model with sY15 for Y14 sulfation. (F) hTPST1-PAP-CCR5 peptide docking model with sY14 for Y15 sulfation.

Figure S9. Continued.

The hTPST1 structures shown in panels A–F are based on the X-ray crystal structure of the hTPST1-PAP-CCR5 peptide complex determined in this study. In panel A, the CCR5 peptide corresponds to the crystallographically observed peptide. In panels B–E, the CCR5 peptides were modeled and energy-minimized using the Molecular Operating Environment (MOE; Chemical Computing Group). In panel F, the CCR5 peptide and the side chain of Lys119 in protomer-B of hTPST1 were modeled and energy-minimized using MOE. Molecular graphics were prepared using PyMOL.

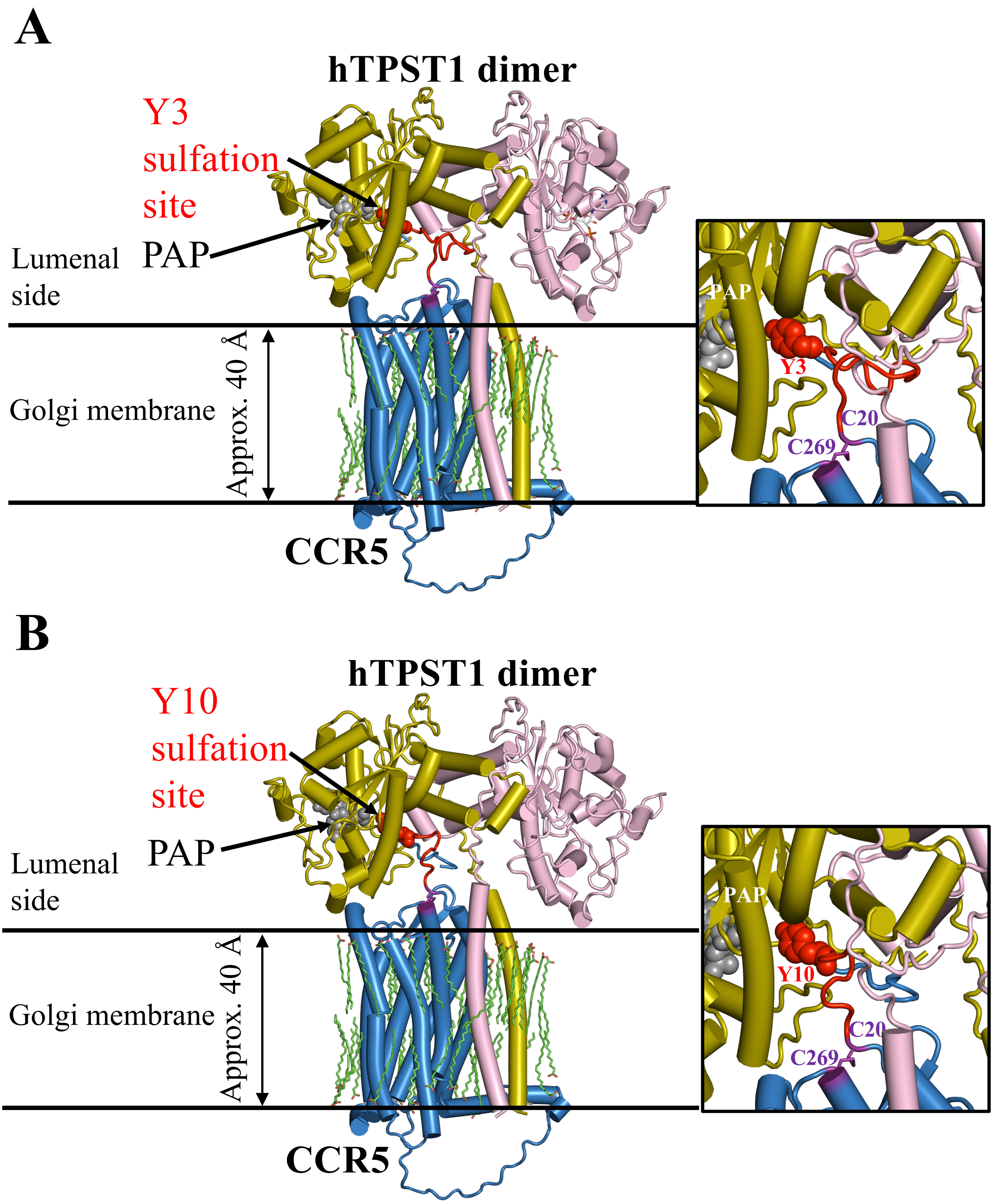

Figure S10. Full-length hTPST1-PAP-CCR5 docking models for Y3 and Y10 sulfation.
Protomer-A and protomer-B of hTPST1 are shown as helix-cylinder models in olive and light pink, respectively. CCR5 is shown with cylindrical helixes in sky blue, and conserved cysteine residues C20 and C269 are shown as purple stick models. PAP is depicted as gray sphere model. Oleic acid lipids (membrane mimetic) are shown as green stick models. The Golgi-mimetic membrane plane is indicated by a black line, and the approximate membrane thickness is ~40 Å. (A) Full-length hTPST1-PAP-CCR5 docking model for Y3 sulfation. The segment between Y3 and C20 (Y3–C20) is highlighted in red and a close-up view is shown at right. (B) Full-length hTPST1-PAP-CCR5 docking model for Y10 sulfation. The segment between Y10 and C20 (Y10–C20) is highlighted in red and a close-up view is shown at right.

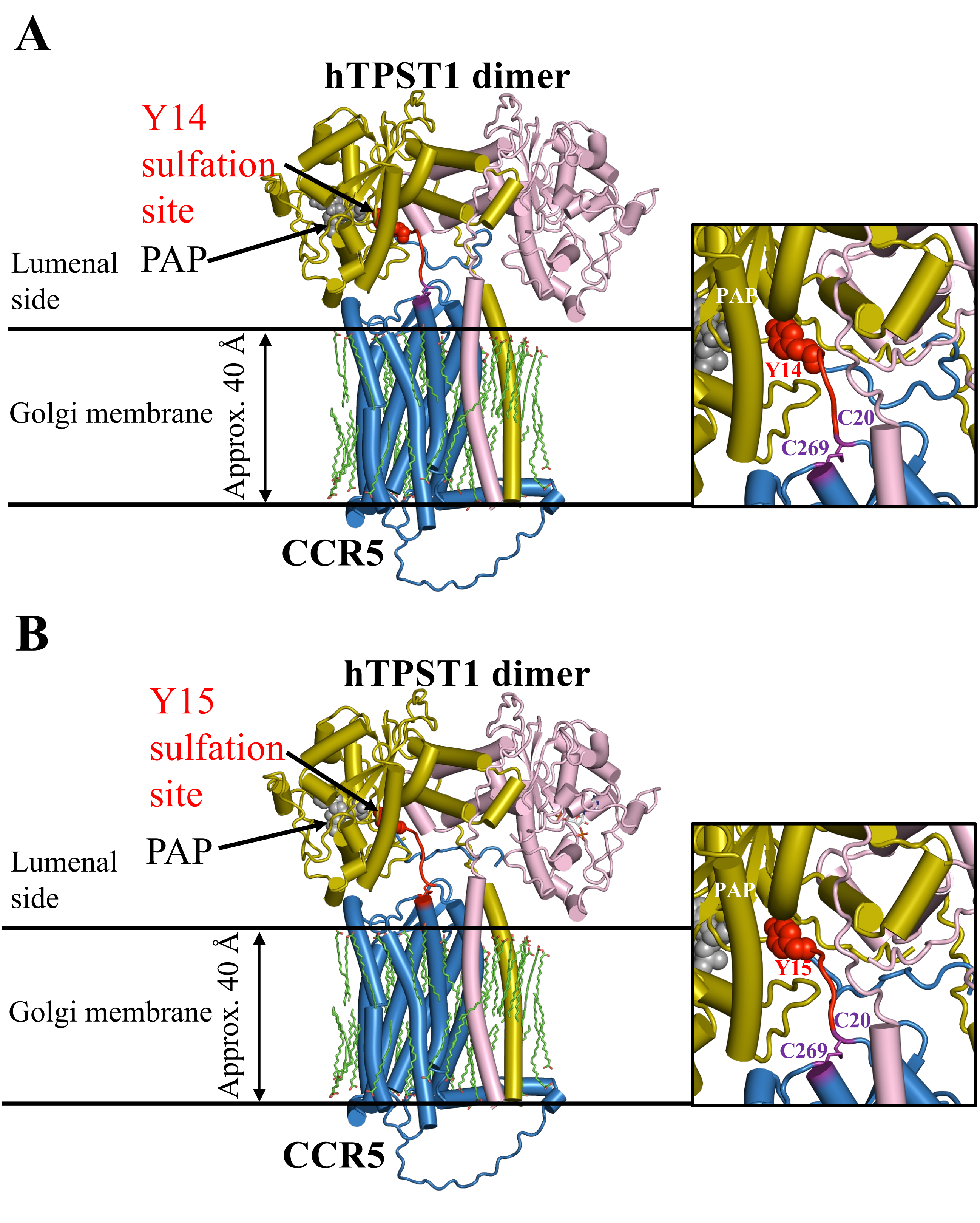

Figure S11. Full-length hTPST1-PAP-CCR5 docking models for Y14 and Y15 sulfation. Protomer-A and protomer-B of hTPST1 are shown as helix-cylinder models in olive and light pink, respectively. CCR5 is shown with cylindrical helixes in sky blue, and conserved cysteine residues C20 and C269 are shown as purple stick models. PAP is depicted as gray sphere model. Oleic acid lipids (membrane mimetic) are shown as green stick models. The Golgi-mimetic membrane plane is indicated by a black line, and the approximate membrane thickness is ~40 Å. (A) Full-length hTPST1-PAP-CCR5 docking model for Y14 sulfation. The segment between Y14 and C20 (Y14–C20) is highlighted in red and a close-up view is shown at right. (B) Full-length hTPST1-PAP-CCR5 docking model for Y15 sulfation. The segment between Y15 and C20 (Y15–C20) is highlighted in red and a close-up view is shown at right.

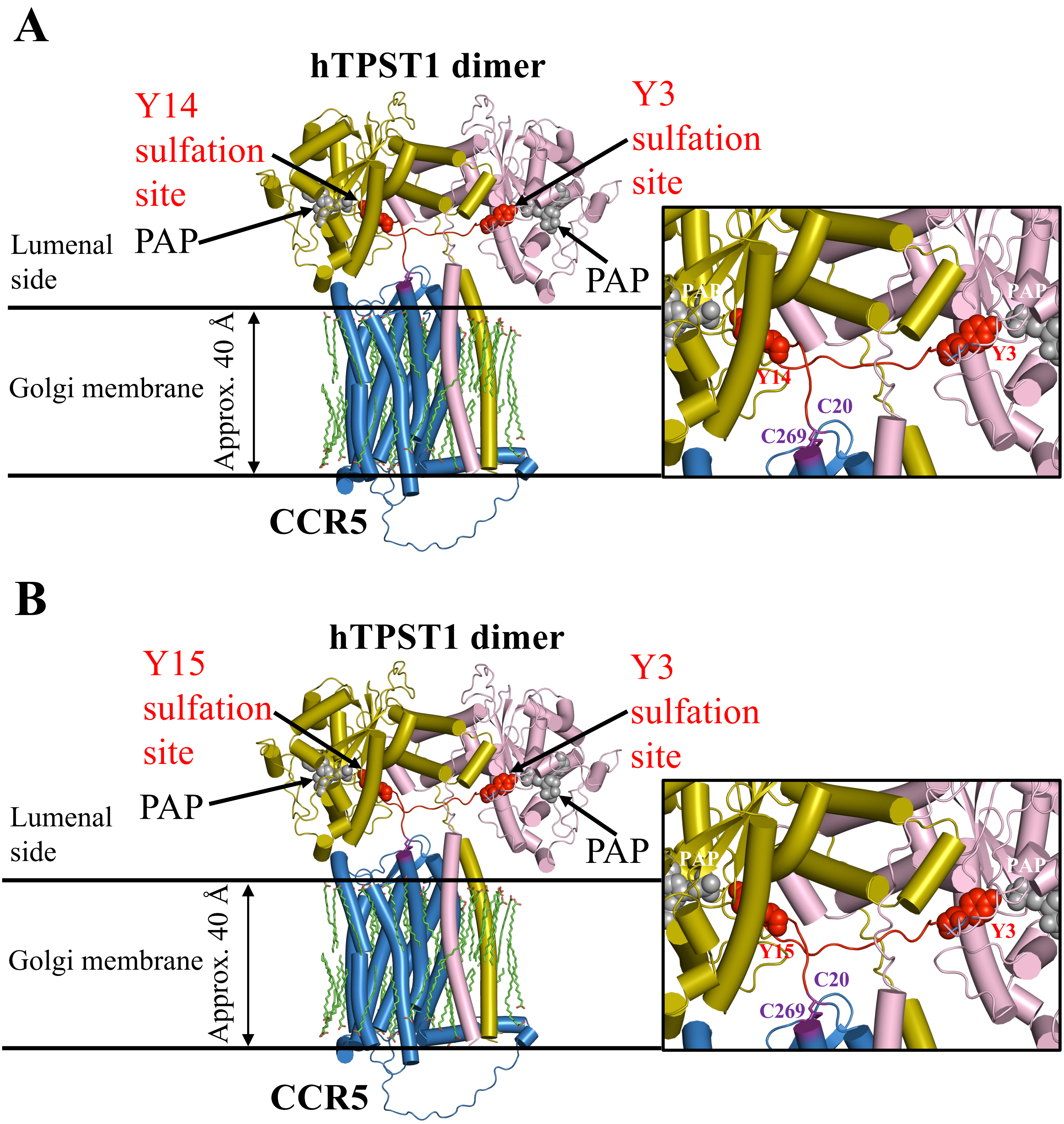

Figure S12. Full-length hTPST1-PAP-CCR5 docking models for Y3 and Y14/Y15 dual sulfation. Protomer-A and protomer-B of hTPST1 are shown as helix-cylinder models in olive and light pink, respectively. CCR5 is shown with cylindrical helixes in sky blue, and conserved cysteine residues C20 and C269 are shown as purple stick models. PAP is depicted as gray sphere model. Oleic acid lipids (membrane mimetic) are shown as green stick models. The Golgi-mimetic membrane plane is indicated by a black line, and the approximate membrane thickness is ~40 Å. (A) Full-length hTPST1-PAP-CCR5 docking model for Y3 and Y14 dual sulfation. The segment between Y3 and C20 (Y3–C20) is highlighted in red and a close-up view is shown at right. (B) Full-length hTPST1-PAP-CCR5 docking model for Y3 and Y15 dual sulfation. The segment between Y3 and C20 (Y3–C20) is highlighted in red and a close-up view is shown at right.

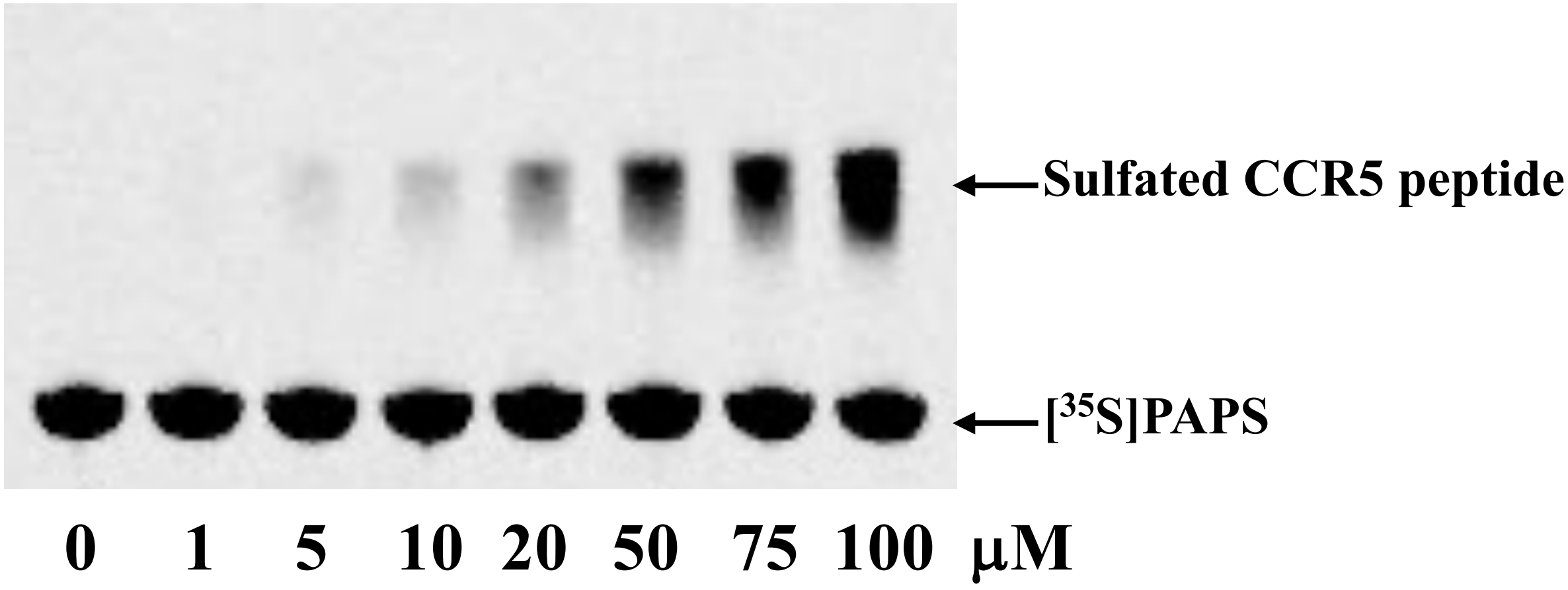

Figure S13. Representative TLC analysis of CCR5 peptide sulfation

Sulfotransferase reactions containing 0, 1, 5, 10, 20, 50, 75, and 100 μM CCR5 peptide and 4.0 μg hTPST1 were analyzed by TLC. Numbers below each lane indicate the CCR5 peptide concentration. The lower bands correspond to [^{35}S]PAPS, and the upper bands correspond to sulfated CCR5 peptide.
